# ‘Fly to a Safer North’: Distributional Shifts of the Orchid *Ophrys insectifera* L. Due to Climate Change

**DOI:** 10.3390/biology11040497

**Published:** 2022-03-24

**Authors:** Martha Charitonidou, Konstantinos Kougioumoutzis, Maria Chara Karypidou, John M. Halley

**Affiliations:** 1 Laboratory of Ecology, Department of Biological Applications & Technology, University of Ioannina, 45110 Ioannina, Greece; jhalley@uoi.gr; 2 Laboratory of Botany, Department of Biology, University of Patras, 26504 Patras, Greece; kkougiou@aua.gr; 3 Department of Meteorology and Climatology, School of Geology, Faculty of Sciences, Aristotle University of Thessaloniki, 54124 Thessaloniki, Greece; karypidou@geo.auth.gr

**Keywords:** climate change, fly orchid, *Ophrys*, Orchidaceae, orchid distribution, range contraction, range shift, species distribution models (SDMs)

## Abstract

**Simple Summary:**

Climate change is one of the major threats to plant diversity and is expected to force species distributions into latitudinal or altitudinal shifts. The complex biology of orchids, and their many interactions with other organisms, increases their vulnerability in a changing climate. This study focuses on how climatic alterations will affect the distribution of the fly orchid (*Ophrys insectifera* L.), one of the most well-known and distinctive *Ophrys* species in Europe, using models that predict the species range changes in the future, based on environmental factors. The orchid’s environmentally suitable area is projected to shift northwards but downhill in the future, experiencing a moderate overall range contraction. More specifically in near- and long-term future, it is expected to be lost in South Europe, especially from the Balkans, while it will gain areas in North Europe, with the UK, Scandinavia, and the Baltic countries being among the winners. These results, although conservative since they are based only on abiotic variables, provide useful insights on the fly orchid’s response to future climatic change, and can serve as a basis for further studies on a finer scale.

**Abstract:**

Numerous orchid species around the world have already been affected by the ongoing climate change, displaying phenological alterations and considerable changes to their distributions. The fly orchid (*Ophrys insectifera* L.) is a well-known and distinctive *Ophrys* species in Europe, with a broad distribution across the continent. This study explores the effects of climate change on the range of *O. insectifera*, using a species distribution models (SDMs) framework that encompasses different climatic models and scenarios for the near- and long-term future. The species’ environmentally suitable area is projected to shift northwards (as expected) but downhill (contrary to usual expectations) in the future. In addition, an overall range contraction is predicted under all investigated combinations of climatic models and scenarios. While this is moderate overall, it includes some regions of severe loss and other areas with major gains. Specifically, *O. insectifera* is projected to experience major area loss in its southern reaches (the Balkans, Italy and Spain), while it will expand its northern limits to North Europe, with the UK, Scandinavia, and the Baltic countries exhibiting the largest gains.

## 1. Introduction

During the last few decades, abrupt climatic changes have induced considerable shifts in the spatiotemporal climatic regimes across the globe [1], with strong effects on all levels of biodiversity already being reported [2,3,4,5,6,7]. Several studies have shown that many organisms will or have already experienced significant range shifts due to climate change, with a poleward or uphill direction. Although the majority of species observed to shift their distributions are abiding to this pattern, there are some that migrate southwards or downhill, showing a wide variety of range alterations [4,5,8]. On the other hand, organisms that fail to migrate or adapt may be more vulnerable to extinction [9]. The number of such species is predicted to increase on a global scale [10,11]. However, local extirpations are already widespread for several species [12]. Adding to this, dispersal due to the changing climatic conditions will not help them avoid extinction, even when combined with niche shifts [6].

Climate change has been listed as a potential threat for plants [13]. The main influence comes from changes in climatic factors like temperature and precipitation that play an important role for the plants’ life cycle [2,3], leading to altered phenologies, mismatches on their interactions with other organisms (e.g., pollinators), and distribution area changes [11,13,14,15]. Global warming seems to force plant species to shift their ranges in latitudinal and/or elevation gradients, in search of more favorable climatic conditions [4,16], while in certain cases, it can even act as an amplifier of their vulnerability to extinction [13,17,18,19]. In both hemispheres, plant species tend to migrate towards the poles, while in altitudinal gradient, they shift mostly uphill (e.g., [4,13,16,20,21]), with exceptions of species following opposite directions existing for both patterns (i.e., towards the equator and downhill, respectively (see [4,5]).

Like all plants, orchids are affected by climatic changes, and their response to those alterations has already been studied. Cases of phenological shifts have been reported [7,22,23,24,25] discussing not only changes in orchid flowering patterns, but also the impact of global warming on orchid-pollinator interactions. In addition, numerous studies have been published about the effects of climate change on the distribution of orchids (e.g., [26,27,28,29,30]), reporting different levels of area change, from low to severe range contractions or even expansions. As for the range shifts, orchids seem to follow the general poleward and uphill trends as a general rule (e.g., [31,32], although there may be some contradictory patterns at a local or regional level [33]. Both temperature and precipitation play an important role in driving distribution changes in orchids (e.g., [33,34,35,36,37]). Additionally, orchid species will be affected by climatic fluctuations in these quantities, and climatic models foresee major increases in the variability of both these factors [38,39].

One of the concerns regarding species’ distributions, is that species may be driven either into regions that are unfavorable or very limited in their extent (e.g., [10]). Orchidaceae are one of the families that are under-assessed regarding their vulnerability to extinction [40]. They could face especially significant losses to their distributions and associated population declines [41,42,43], because of the complexity of their life cycle and their symbiotic dependence on other organisms that could act as an amplifier of extinction probability. Consequently, orchids could face greater risks from global warming than in other families [44].

The fly orchid (*Ophrys insectifera* L.) is one of the most distinctive *Ophrys*, serving as the type species of the genus. It is characterized by a wide distributional range throughout the continent [45,46], and it can be found in a variety of habitats over a broad altitudinal extent [45,47]. However, in most cases, it occurs in areas with increased soil moisture (damp soils), and always in full sun or semi-shaded sites (see [47,48,49]). Thus, despite its widespread occurrence in Europe, there is cause for concern regarding the conservation of this species in the future. The scope of this paper is to investigate whether such concerns have a reasonable basis. For this purpose, the study focuses on the effects of climate change on the distribution of *O. insectifera* in the upcoming decades, using a species distribution models (SDMs) framework under different combinations of global circulation models (GCMs), share socioeconomic pathways (SSPs), and future time periods.

## 2. Materials and Methods

### 2.1. Study Species

**Figure 1:**
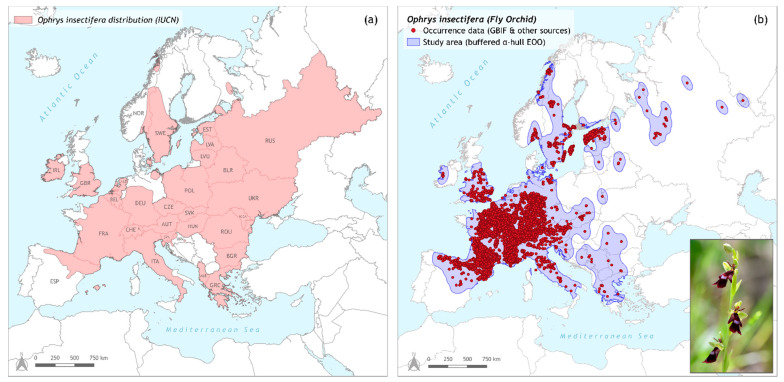
(**a**) *Ophrys insectifera* distribution map, compiled with data downloaded from IUCN [53], (**b**) map including the total occurrence data for *O. insectifera* retrieved from GBIF and other sources, and the study area defined as a buffered α-hull extent of occurrence (EOO). Both maps are designed in QGIS v.3.18 ‘Zürich’, using ETRS89—Lambert Conformal Conic Coordinate Reference System. Photo of the species taken in June 2019 at Mt. Tzoumérka (Epirus, NW Greece).

The fly orchid (*Ophrys insectifera* L.) was first observed and described in the 1750s by Linnaeus, during his trip to the Baltic islands [50], and serves as the type species of the genus *Ophrys*. It is one of the most distinctive bee orchids, with its lax inflorescence bearing the characteristic fly-resembling flowers, that, in contrast to their appearance, are pollinated by male digger wasps of the genus *Argogorytes* (*A. mystaceus* and *A. fargeii*) [51]. It is a tuberous perennial orchid, with a height varying from (12-)15 to 50 cm, which can be found in bloom during May–July (depending on the region), in damp, full sun/semi-shade sites, in a variety of habitats (from woodlands and forest edges to fens and grasslands), and in a broad altitudinal range (0–1700 m a.s.l.) [45,47]. *O. insectifera* is a species native to Europe, and in contrast to the mainly Mediterranean range of other bee orchids, it has an extended distribution throughout the continent [45]. Extending from Ireland and Northern Spain to Ukraine (West–East Axis), and from Scandinavia and the Baltic countries to Italy and Northern Greece (North–South Axis) [46], its distribution center lies in Central Europe. However, marginal occurrences can be found in Russia and Northern Norway, thus characterizing *O. insectifera* as a rather temperate species and the northernmost bee orchid [47] (Figure 1a). Despite its wide range throughout the European continent, its populations can be locally dense or even locally rare, with only scattered occurrences [52].

### 2.2. Species Occurrence Dataset and Coordinate Thinning Procedures

Occurrence data for *Ophrys insectifera* were downloaded from the Global Biodiversity Information Facility (GBIF) database (a total of 63,574 occurrences) [54]. In this dataset, available occurrences for Greece (20 occurrences; ‘Orchids of Greece’ project database, Assist. Prof. Spyros Tsiftsis pers. comm.), Serbia (seven occurrences; [55]), Bulgaria (six occurrences; [56]), and Romania (four occurrences; Mihai Bobocea pers. comm.) were added. The original occurrence dataset for *O. insectifera* derives from the available data from GBIF. However, as shown in Figure 1a, the distribution of the species contains other countries as well (e.g., in Eastern Europe and the Balkans), for which there are no data in the GBIF repository. This issue could question the reliability of the predictions for the species’ range. Nevertheless, it was tackled to some extent with the aforementioned occurrence data additions, and by setting the species distribution area to be equal to the extent of occurrence (EOO) as proposed by guidelines from the International Union for Conservation of Nature (IUCN) [57]. This extent of occurrence was used as a proxy for the species distribution [58] and was calculated using the R-package ‘ConR’ 1.3.0 [59] with the alpha-hull method, adding a buffer around the calculated extent to cover all occurrence data. 

All non-georeferenced occurrences were removed from the dataset, as well as any pre-1970 data in order to match the temporal resolution of WorldClim v2.1 baseline data [60]. All points with coordinate uncertainty > 9.25 km were also removed, so as to be in line with the resolution of the selected environmental variables (see Section 2.3). Moreover, the functions ‘clean_coordinates’ of the ‘CoordinateCleaner’ 2.0.18 R package [61] and ‘elimCellDups’ function from the ‘enmSdm’ 0.5.3.3 R package [62] were used to further clean the occurrence dataset and eliminate any duplicate records, respectively. 

The remaining occurrence data were spatially thinned using the R package ‘spThin’ 0.1.0 [63] following Robertson et al. [64], resulting in a dataset of 3914 records (hereafter GeoThin). However, according to Varela et al. [65], environmental thinning may lead to improved model performance. To that end, spatially thinned occurrences were further thinned based on the representative and uncorrelated environmental variables occurring in the study area (see Section 2.3), following Varela et al. [65] (and the code provided at: https://github.com/SaraVarela/envSample; last accessed on 26 February 2022). After environmental thinning, the second occurrence dataset consisted of 1802 records (hereafter, EnvThin).

### 2.3. Environmental Data

Current and future climatic data (minimum temperature, maximum temperature, average temperature, precipitation, and 19 bioclimatic variables) were downloaded from the WorldClim v2.1 database [60] at a 5 arc minutes resolution. Regarding future projections, data from the Coupled Model Intercomparison Project—Phase 6 (CMIP6) were obtained for two time slices, 2070 (timeframe 2061–2080, representing ‘near-term future’) and 2090 (timeframe 2081–2100, representing ‘long-term future’), for three GCMs (BCC-CSM2-MR [66], MIROC-ES2L [67], MRI-ESM2-0 [68]), and four different SSP scenarios [69] (SSP1-2.6, SSP2-4.5, SSP3-7.0, SSP5-8.5). Extra-terrestrial solar radiation and 16 additional bioclimatic variables were constructed for all time-slices using functions from the R package ‘envirem’ 2.2 [70]. Elevation data were downloaded via the CGIAR-CSI data-portal [71], and five topographical variables (slope, aspect, heat load index, topographic position index and terrain ruggedness index) were then estimated, with the R packages ‘raster’ 3.3.13 [72] and ‘spatialEco’ 1.2-0 [73]. Soil variables were extracted from the SoilGrids 2.0 database [74] via Web Coverage Services (WCS) in QGIS v.3.18.0 ‘Zürich’ [75] at a 5 arc minutes resolution. The function ‘vifcor’ from the R package ‘usdm’ 1.1.18 [76] was used to assess multicollinearity. From the initial set of 52 chosen predicting variables, 20 did not show any collinearity problems (Spearman rank correlation < 0.7 and VIF < 10; [77]) and were, thus, included in the analyses (Table S1). 

### 2.4. Species Distribution Models

The realized climatic niche of *Ophrys insectifera* was modeled by combining the occurrences’ dataset with current environmental predictors in an ensemble modelling scheme, to reduce model algorithm uncertainty [78,79]. This process was followed for both thinning procedures (GeoThin and EnvThin occurrence datasets).

Fine-tuned SDMs were fitted based on four different algorithms: random forests (RF), boosted regression trees (BRT), Bayesian additive regression trees (BART) and maximum entropy (MaxEnt), via the R packages ‘SDMtune’ 1.1.4 [80], ‘embarcadero’ 1.2.0.1003 [81] and ‘ENMeval’ 0.3.1 [82], respectively. Before the procedure of model fitting, spatial cross-validation was applied in order to reduce spatial autocorrelation [83], by spatially partitioning the dataset in four blocks, using the function ‘get.block’ from the R package ‘ENMeval’ 0.3.1 [82]. Regarding the algorithms RF and BRT, models’ hyperparameters were fine-tuned using the functions ‘reduceVar’ and ‘optimizeModel’ of the ‘SDMtune’ R package [80]. There, any variables with low permutation importance (nperm = 100; <5%) were sequentially removed until the optimum Jackknife test TSS was reached, and afterwards, the hyperparameter combination of the best performing model based on test TSS was identified. For BART, fine-tuning process was applied using the functions ‘bart.step’ and ‘retune’ as described in Carlson et al. [84]. For MaxEnt, the combinations between linear, quadratic, and hinge feature classes were explored, since they lead to more comprehensive and better-performing models [85]. The regularization multipliers ranged from 1 to 10 with a step value of 0.1, resulting in 600 candidate models. Optimal MaxEnt model settings were identified based on threshold-dependent (i.e., omission rate) evaluation metrics, in order to prevent overfitting and improve model transferability, as model predictions based on information criteria lead to oversimplified models with low predictive performance [86]. 

Pseudo-absences (PAs) for *O. insectifera* were generated following the recommendations of Barbet-Massin et al. [87] and Liu et al. [88]: 30,000 background points were randomly sampled within the study area (defined in Section 2.2), since poor background sampling may lead to a truncated environmental response [89]. 

The prediction capability of each one of the models was evaluated using a selection of discrimination and calibration metrics. In order to avoid any misleading result by relying to a single metric [90,91,92], four discrimination (AUC, AUC-PR, Sørensen’s index, TSS) and three calibration (Brier score, Cohen’s Kappa, Continuous Boyce Index (CBI)) metrics were selected for the evaluation [93,94,95,96,97,98]. The aforementioned metrics were calculated using functions from the ‘CalibratR’ 0.1.2, ‘DescTools’ 0.99.40, ‘ecospat’ 3.2, ‘enmSdm’ 0.5.3.2, ‘Metrics’ 0.1.4, ‘MLmetrics’ 1.1.1 and ‘modEvA’ 2.0, R packages [62,99,100,101,102,103,104]. 

Variable importance for each model was estimated via the functions ‘varImp’ (nperm = 1000), ‘varimp’ and ‘var.importance’ from the ‘SDMtune’, ‘embarcadero’ and ‘ENMeval’ R packages, respectively.

The potential distribution of *O. insectifera* was projected under current and future climate conditions, for all algorithms separately (regarding MaxEnt, the ‘clogclog’ output was used, which is analogous to the occurrence probability predictions from the remaining algorithms [85]), as well as via an ensemble model framework [79], based on calibrated models with TSS ≥ 0.6 (to avoid poorly calibrated ones). The contribution of each model to the ensemble forecast was weighted according to its TSS score. Five ensemble methods were selected (median, mean, weighted mean, committee average and PCA-based), to tackle performance uncertainty [105], and from these the best ensemble model was selected based on TSS and Sorensen’s index [92].

The resulting habitat suitability maps were transformed to binary based on the metric that maximizes the sum of sensitivity and specificity [96,106,107] and the one that maximizes Sorensen’s index as suggested by Leroy et al. [92]. Afterwards, those were compared to the binary maps obtained for each GCM and SSP. As a conservative approach, the suitability of any cells that had non-zero values in the clamping mask was set to NA [108]. Regarding models produced by ‘embarcadero’, the suitability of any cells that had an uncertainty value equal to or higher than the 90% of the cells was set to NA.

Finally, function ‘BIOMOD_RangeSize’ of the R package “biomod2” 3.3.7 [109] was used to assess the projected range change of the species, for the individual and ensemble models for both thinning procedures. *O. insectifera*, like all orchids, produces numerous dust-like seeds, with extremely low weight, that theoretically can “travel” for long distances [110]. To that end, for this analysis an unlimited dispersal capacity was assumed for the species range.

### 2.5. Distribution Shift in Latitudinal and Altitudinal Gradient

In order to test if the distribution of *Ophrys insectifera* shifts latitudinally in the future, the median centroids of distributions of current and future time slices were calculated based on the binarized habitat suitability from the ensembles of both thinning procedures, and for all GCMs/SSPs, using the function ‘st_centroid’ of the R package ‘sf’ 1.0.5 [111]. A distance matrix between current and future centroids was calculated for all cases (near- and long-term future for EnvThin and GeoThin ensemble) from the homonymous toolbox in QGIS v.3.18.0 ‘Zürich’ [75] software. Moreover, to examine the altitudinal shift of the species, elevation data from the CGIAR-CSI data-portal were used in order to extract the mean altitude for the current and future projections, for all combinations of thinning procedures, GCMs and SSPs. Finally, for both types of shifts, non-parametric tests were used, in order to check for any statistically significant difference (functions ‘kruskal.test’ and ‘pairwise.wilcox.test’ of the R-package ‘stats’).

### 2.6. Response of the Most Important Variable in Locations of Interest

In order to investigate the effect of the most important variable on the distribution of *Ophrys insectifera*, two locations were selected from the species’ range that represent areas of consistent loss and gain and are congruent under all studied combinations of models and scenarios. Three sets of simulations were utilized for each one of the selected GCMs: the historical simulation (1986–2014), SSP1-2.6 (best-case scenario), and SSP5-8.5 (worst-case scenario) for the period 2015–2100. Simulations for MIROC-ES2L and MRI-ESM2-0 were retrieved from the Copernicus Climate Data Store (https://cds.climate.copernicus.eu/; accessed on 31 January 2022) using the “cdsapi” python tool (https://pypi.org/project/cdsapi/; accessed on 31 January 2022), while BCC-CSM2-MR simulations were retrieved from the Earth System Grid Federation (https://esgf-data.dkrz.de/projects/esgf-dkrz/; accessed on 31 January 2022) using bash scripts. Data were analyzed in their native horizontal resolution, and the required variable is calculated from the original data, following the definition by [58], for every year during the period 1986–2100 for each GCM and SSP. Time series were extracted without smoothing, based on the grid point that was the nearest neighbor to each one of the two selected locations. The processing of the raw files was performed using Climate Data Operators (https://code.mpimet.mpg.de/projects/cdo/; accessed on 31 January 2022), while data were plotted using the R package ‘ggplot2’ [112].

## 3. Results

All models—apart from MaxEnt—performed sufficiently well (TSS: 0.747 ± 0.232; AUC: 0.895 ± 0.120; PRAUC: 0.782 ± 0.317; Cohen’s Kappa: 0.598 ± 0.318; CBI: 0.994 ± 0.009; Sorensen’s index: 0.794 ± 0.011; Brier Score: 0.059 ± 0.049; see Table S2). Ensemble models performed equally well (median AUC: 0.994 ± 0.003; Brier score: 0.000 ± 0.000; CBI: 0.978 ± 0.043; Sorensen’s index: 0.774 ± 0.052; TSS: 0.947 ± 0.028; see Table S3).

Among all the response variables, temperature seasonality had the highest contribution for the majority of combinations of thinning procedures and algorithms, followed by the Thornthwaite Aridity Index. Exceptions to this were the case of EnvThin-BART, where Thornthwaite Aridity Index was the most important variable followed by Temperature seasonality, and the ones of EnvThin-RF, GeoThin-RF and GeoThin-BRT, where elevation and Precipitation Seasonality were the second most important variables (Table S4). 

**Figure 2:**
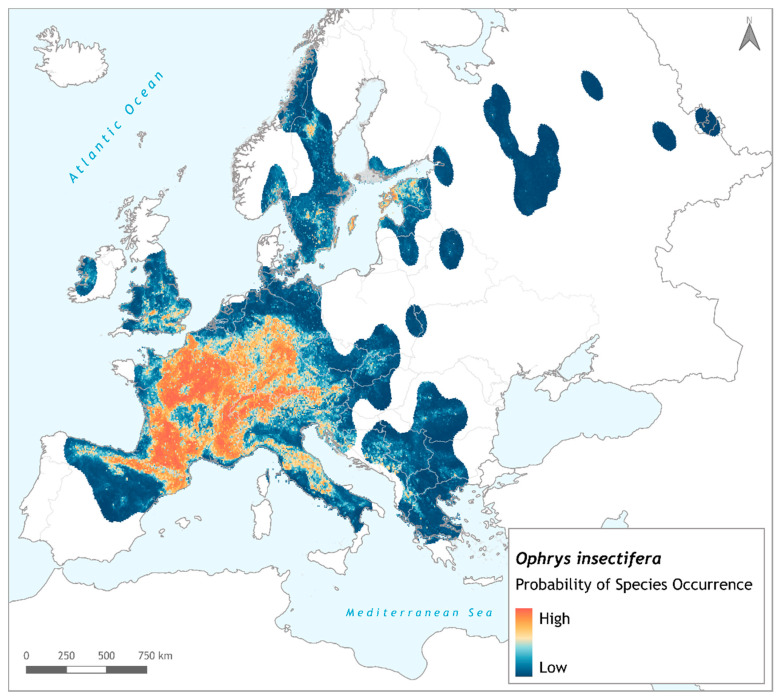
Current habitat suitability map for *Ophrys insectifera*, for the ensemble model using the environmental thinning procedure. Map is designed in QGIS v.3.18 ‘Zürich’, using ETRS89—Lambert Conformal Conic Coordinate Reference System.

The resulting habitat suitability maps that were similar for the ensemble of both thinning procedures (Figure 2 and Figure S2) were converted into binary maps, and then compared to the binary maps obtained for each GCM, SSP scenario, time-slice, and thinning procedure. Since the outcome of the ensemble modeling for the current and future potential distribution of *Ophrys insectifera* was largely similar across all combinations of thinning procedures and future scenarios, results for the EnvThin ensemble were selected to be shown in-text. In addition, for future predictions, only SSP1-2.6 and SSP5-8.5 are presented in-text, as best-case and worst-case scenario, respectively.

**Figure 3:**
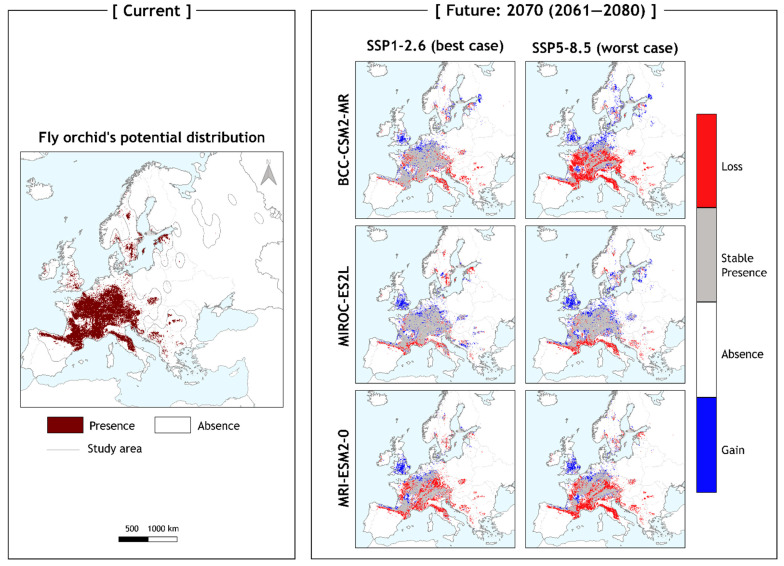
Current and future potential distribution maps for *Ophrys insectifera* EnvThin ensemble model. Left-hand panel: red-brown coloring indicates the cells the species currently potentially occupies. Each map is showing the transition from the present time-period to each respective GCM and SSP combination. Right-hand panel: future potential distribution maps of 2070 for the combinations of three GCMs (BCC, MIROC, and MRI) and two SSPs (SSP1-2.6 and SSP5-8.5) as ‘best’ and ‘worst’ case scenario. Grid cells with red coloring indicate the areas where the species is currently present but will not be in the future. Grey coloring represents cells where the species currently occupies and will continue to occupy in the future. White stands for the cells where the species is not currently present will not be in the future, while blue grid cells indicate the areas where the species is not currently present but will occupy in the future. All maps are designed in QGIS v.3.18 ‘Zürich’, using ETRS89—Lambert Conformal Conic Coordinate Reference System.

*Ophrys insectifera* is projected to experience moderate overall contraction of its environmentally suitable area in the near-term future (mean contraction in the worst-case scenario of the presented EnvThin ensemble: 15.59%—2070; mean current occurrence lost for the worst-case scenario of the presented EnvThin ensemble: 27.65%—2070; Table 1). However, the species is expected to face large changes across its distribution. A considerable area loss is observed (mean expected loss: 38.48% for the worst-case future scenario of EnvThin ensemble in 2070; Table 1), mainly at the southern parts of the species’ distribution, as well as at its west and east edges (Figure 3). *O. insectifera* is also anticipated to show an increase of environmentally suitable areas in the near future (mean expected gain: 22.9% for the worst-case scenario of EnvThin ensemble in 2070; Table 1). This gain is observed mainly at the leading edge of the distribution, with some sporadic suitable areas’ increase at regions on both sides and close to the distribution centre (Figure 3). Results for long-term future (2090) show similar trends for the range changes of the species. Contraction of environmentally suitable areas is expected to be higher than in 2070 (mean contraction for the EnvThin ensemble in 2090: 27.51%; mean current occurrence lost for the worst-case scenario of the presented EnvThin ensemble: 40.12%—2090; Table 1). Loss of currently occupied areas is expected to be larger than in 2070 (mean expected loss: 48.87% for the worst-case scenario of EnvThin ensemble in 2090; Table 1), and observed at the trailing edge of the distribution in the south (Figure S2). Area gain is slightly lower than in 2070 (21.35%; Table 1) and located in the same areas at the northern parts of the distribution (Figure S2). The results are consistent throughout all combinations of GCMs, SSPs and future time slices, for both EnvThin and GeoThin ensemble models (see Figures S3–S8 and Tables S5 and S6).

**Figure 4:**
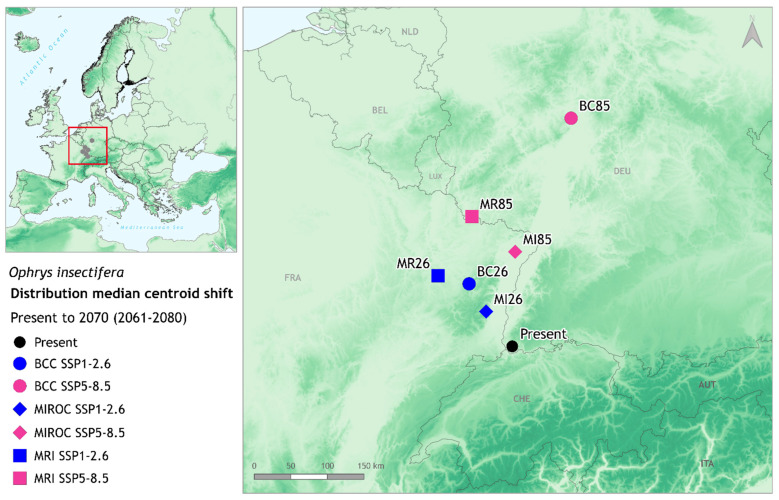
Median centroids for current and future projected distributions of *Ophrys insectifera*, for the EnvThin ensemble in 2070 time slice. Black point represents the current distribution’s median centroid, the blue points stand for the future best-case scenario (SSP1-2.6), and the magenta points for the future worst-case scenario (SSP5-8.5). GCMs differentiate by shape: BCC—circle, MIROC—diamond, MRI—square. Map is designed in QGIS v.3.18 ‘Zürich’, using ETRS89—Lambert Conformal Conic Coordinate Reference System.

The northwards shift of the species can be observed more easily by looking at the centroid movement. The centroids of all projected future environmentally suitable areas of *Ophrys insectifera* for the selected ensemble appear to be always lying to the north and mainly northwest of the current centroid, with very few exceptions, e.g., BC85 centroid in the presented ensemble, that is placed to the northeast of the current (Figure 4 and Figure S10). The distance between the current and future centroids depended on the combination of GCM and SSP, and in the presented EnvThin ensemble for 2070 varied from 59.6 to 323.5 km (MI26 and BC85 respectively; Table S9). For 2090, the centroids for all combinations of GCMs and SSPs show similar patterns (Figures S9 and S11), with the distance between the current and future centroids being even larger (85.7 to 759.2 km, for MI26 and BC85 respectively; Table S9). Results are consistent among all combinations of GCMs, SSPs and future time slices for both EnvThin and GeoThin ensemble models (Figures S12–S15 and Table S10).

The results for the altitudinal shift test showed a moderate downhill movement for all different combinations of GCMs and SSPs of future projections for both 2070 and 2090 (Table 2 and Table S7; 2070 Kruskal-Wallis test x^2^ = 863.38, df = 6, *p* < 0.001; 2090 Kruskal-Wallis test x^2^ = 3504.2, df = 6, *p* < 0.001). The species can currently be found at a mean altitude of 542.6 m a.s.l. for the presented ensemble, while in future it is expected to be found in areas with a mean altitude lower by ca. 100–140 m for the worst-case scenario of 2070 and 2090 respectively. Similar results have been observed for all combinations of GCMs, SSPs and future time slices for the GeoThin ensemble (Table S8).

**Figure 5:**
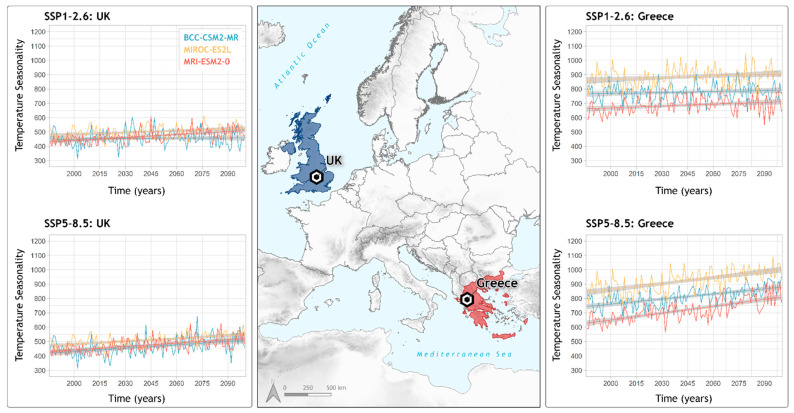
Temperature seasonality variation for two selected locations of *Ophrys insectifera* distribution over the time period 1986–2100. UK (in blue) represents an area of range gain, while Greece (in red) an area of range loss. Two shared socioeconomic pathways (SSPs) are presented, as best-case and worst-case scenarios (SSP1-2.6 and SSP5-8.5, respectively). In all panels, each line represents a global circulation model (GCM): BCC-CSM2-MR (cyan line); MIROC-ES2L (yellow line); MRI-ESM2-0 (coral red line).

Figure 5 shows the time series for temperature variability (yielded as the most important variable in SDMs) for the two selected locations, Greece and the UK, at the southern and northern reach of *Ophrys insectifera*, respectively. As mentioned in Section 2.6, these two locations were selected as examples for comparison purposes, since they consistently exhibit high rates of area loss (Greece) and gain (UK) in all future projections. Temperature seasonality in Greece displays a consistent increase with a high intra-annual variability for all GCMs, which is maximized for the worst-case scenario. On the other hand, GCMs for the UK exhibit a considerable consensus compared to Greece. Over this location, temperature seasonality also shows an increasing trend, however the magnitudes of change are significantly smaller than in Greece.

## 4. Discussion

On the basis of results obtained in this study, the environmentally suitable area of *Ophrys insectifera* is expected to shift northwards. Its future centroid will shift by 59.6 to 323.5 km (EnvThin ensemble’s MIROC SSP1-2.6 and BCC SSP5-8.5, respectively; Table S9) from its current location, which lies close to the tripoint of France, Germany and Switzerland (Figure 4). This northward shift is robust under all investigated climatic models, scenarios, and future time frames (Figures S9–S15). By contrast, there is no consistent direction, westward or eastward in the forecasts for the environmentally suitable areas. This migration pattern of the fly orchid is in line with the prevailing trend anticipated for plants in general (see [4,13,16]), and for other orchid species in Europe, including *Cypripedium calceolus* [32], *Epipactis helleborine* [35], *Himantoglossum hircinum* [31,113], *Orchis militaris, O. simia, O. anthropophora* and *O. purpurea* [26]. 

Also expected is a moderate downhill movement (under all investigated climatic models, scenarios and future time frames; Tables S7 and S8). In the presented EnvThin ensemble model *O. insectifera* shows a mean altitudinal difference of 115 m by 2070 and of 265 m by 2090 (Table 2). Although this downhill shift goes against the common uphill pattern, it has been observed before. Lenoir et al. [5] found that 30.9% of forest plant species in Europe are expected to move to lower elevation in the future, while Chen et al. [4] showed that species exhibit high variation of shifts across groups and regions, with ca. 25% of the studied taxa moving downhill, in contrast to the mainstream uphill trend. This may be due to a complex interaction between the altitudinal and latitudinal temperature gradients, that strongly affect the niche patterns in areas of extended elevation shift. 

There have been long-term observations of orchid species in Europe, which report decline in numbers, as well as a systematic contraction of the range in certain European orchids [114,115]. This contraction is expected to intensify due to climatic change. For example, *Epipactis helleborine* is expected to face a decrease of 25–40% of its suitable habitat areas until 2080 [35], *Traunsteinera globosa* is projected to show a future range contraction of 18–32% [29], and *Orchis militaris*, that is anticipated to decrease by up to 61.3% in the future worst-case scenario [26]. *O. insectifera* follows the same trend as other orchids with temperate extent, showing a moderate contraction pattern, for all studied models and scenarios, which will reach 27.5% in the worst-case scenario in the long-term future.

According to Evans et al. [26], as climate continues to change, areas in Northern Europe will become more suitable for orchid species, in contrast to southern European countries that are expected to experience more intense climatic alterations (see also [1]). The aforementioned future changes in the suitable areas of *Ophrys insectifera* are mainly driven by temperature variability, and aridity (see Table S4). Temperature is known to influence orchid distributions and abundance (e.g., [35,37,116]). Although orchids can be tolerant to temperature seasonal changes, large fluctuations can have negative effects on the life cycle and the species’ population dynamics [117]. Soil moisture is also a factor that strongly influences orchid distributions, through its effect on the below-ground stages of the orchids’ life cycle [118]. However, soil moisture, being a factor associated with microclimate [117], cannot be easily included in a large-scale analysis, and thus is absent from available databases. Nevertheless, aridity can serve as a proxy for soil moisture, and thus the distribution changes of the species exhibit considerable dependence on this factor. 

Despite the overall moderate change predicted in this study, *O. insectifera* will exhibit strong patterns of environmentally suitable area loss and gain in certain locations. More specifically, the species will show high percentages of loss at the southern reach of its current extent (Figure 3 and Figures S2–S8). This includes Spain, Italy, and countries of the Balkan Peninsula such as Bulgaria and Greece, from which it will disappear entirely. On the other hand, the northern reach of the species distribution will see large gains of suitable areas. Among the regions projected to show gain in the north of Europe, the Baltic countries, Scandinavia and especially the UK appear as gain hotspots (Figure 3 and Figures S2–S8). *O. insectifera* is the only *Ophrys* with a European temperate distribution that is found in a wide altitudinal range across its extent, but always in sites with increased water presence [45,48], in contrast to other bee orchids, that are adapted to drier conditions occurring in Mediterranean habitats [47]. Larger fluctuations and high increase of temperature seasonality observed in the south of Europe are in contrast to the less intense ones to the north (Figure 5). This, combined with the vast increase of aridity, can explain the projected changes of the species in future decades. This result is also in line with the findings of Thuiller et al. [18], that found high percentages of plant species’ loss in the southern parts of Europe (up to 62.1% in Mediterranean mountainous areas), and high percentages of species’ turnover in Northern Europe (Central Atlantic, Continental and Boreal biogeographic regions).

The anticipated gain of area to the north reach of *O. insectifera* seen in this study is in contrast to trends observed so far. Declines of the species have been observed in the Netherlands, Flanders, Estonia and the UK [114,119,120,121], with an exception coming from Denmark, where the species has been showing an increase the last 30 years [122]. However, such declines from the past to present, probably reflect previous land use change (see [123]). Although the key driver of declines in the future is likely to be climatic, land use change will also play an important role.

On the other hand, the observed loss in southern Europe under all investigated cases can also raise concerns about the conservation of the species’ diversity. More specifically, at the southwest border of *O. insectifera* distribution, there have been described two endemic subspecies, *O.insectifera* subsp. *subinsectifera* in the Spanish Pyrenees, and *O. insectifera* subsp. *aymoninii* in the French Causses region [46,47]. Although they differentiate in a phenological aspect, their genetic differentiation is rather weak [124]. Additionally, at the southeastern limits of the species’ distribution, Greek and Bulgarian populations of *O. insectifera* were genetically distant from others, and were characterized by a unique haplotype, underlining a cryptic diversity within the species [124]. According to this study’s results, areas that currently host this diversity at the trailing edge of the species are projected to be lost in future time slices, under all combinations of models and scenarios of climate change, pointing to an urgent need for conservation of genetic resources.

Despite the favorable performance metrics and the congruence of results between all combinations of models and scenarios, the estimated predictions for *O. insectifera* could be characterized as conservative, and potentially underestimating the actual trends. Among the limitations of SDMs, is the difficulty in modelling the effect of interaction between species, population dynamics and evolution. Orchids are known to depend on symbiotic relationships with other organisms above and below ground for their survival [125,126]. *O. insectifera* is a specialist that relies on two specific digger wasp species (*Argogorytes* spp.) for its pollination [48]. Below-ground, it also relies on interactions with fungi of Tulasnellaceae (at the mature stage) and Ceratobasidiaceae (at the protocorm stage) [127]. These interactions add a level of complexity when dealing with climatic changes; in order for a species to shift its distribution without further adaptations, so should its close partners. An alternative outcome in such a situation is the evolution of new or substantially modified interactions. Evolution driven by climatic change has been stated before for the genus *Ophrys*, that during the Pleistocene exhibited high diversification rates and shifted from wasp- to bee-pollinated [128]. However, it should be noted that this past evolution due to climate change occurred over a much longer time scale compared to the current climatic alterations, where many species will fail to cope with its comparatively rapid pace [19]. As for their population dynamics, orchids have shown great variability of intra- and interspecific growth rates [126], and at the same time show a degree of resilience to environmental changes. Nevertheless, extremes of environmental variability can result to more dramatic population declines, which, combined with orchids’ massive fecundity, can lead to population extirpations [129] and further range changes of orchid taxa. However, such effects are beyond the intended scope of this paper.

## 5. Conclusions

This study explored the effects of climate change on the distribution of the fly orchid (*Ophrys insectifera* L.) in the near- and long-term future, by means of a species distribution models (SDMs) framework, under different combinations of global circulation models (GCMs), share socioeconomic pathways (SSPs), and three different future time periods. According to our findings, *O. insectifera* is expected to face moderate contractions of its environmentally suitable area in the future, with a projected loss at the warm edge of its distribution under all possible cases examined. However, a possible expansion of its environmentally suitable area is observed on its leading edge, while the species is anticipated to move northwards and downhill to cope with the changing environmental conditions.

These results provide a basis for further investigation of the effects of the changing climate on this particular orchid. Additional studies should focus on combining outcome of SDMs with dynamic population models, especially for populations that are anticipated to face major climatic changes, as well as those at the leading edges of the species’ distribution, where gain is expected. Of special importance are issues involving land-use change, since such factors are believed to be behind widely reported declines in orchid abundance in Europe, which contrasts with the increases expected in northern Europe due to climatic shifts. The effect of land use change is also listed among the factors that strongly affect the distributions of orchid species (see [123]), hence, further work should focus on how changes in land use can affect the distribution of *Ophrys insectifera* in future decades.

## Figures and Tables

**Table 1:** Percentages of area loss, gain, overall area change and current occurrences lost for future projections of *Ophrys insectifera*. For the future, 2 time slices, 2070 (2061–2080) and 2090 (2081–2100), are presented for all selected global circulation models (BC: BCC-CSM2-MR, MI: MIROC-ES2L, MR: MRI-ESM2-0) and two shared socioeconomic pathways (SSP1: best-case scenario, SSP5: worst-case scenario). The presented values are for the ensemble model with the environmental thinning procedure.

Time Slice	Transition	GCM	Area Loss (%)	Area Gain (%)	Overall Change (%)	Current Occurrences Lost (%)
2070	Present to SSP1-2.6	BC	20.58	17.45	−3.14	8.27
		MI	16.40	21.12	4.72	10.54
		MR	38.29	14.36	−23.93	22.03
		**Mean**	**25.09**	**17.64**	**−7.45**	**13.60**
	Present to SSP5-8.5	BC	51.42	23.89	−27.54	38.46
		MI	20.03	29.36	9.33	13.37
		MR	44.00	15.44	−28.55	31.13
		**Mean**	**38.48**	**22.90**	**−15.59**	**27.65**
2090	Present to SSP1-2.6	BC	20.07	15.03	−5.04	8.99
		MI	16.13	22.91	6.78	10.27
		MR	31.64	13.25	−18.39	16.98
		**Mean**	**22.61**	**17.06**	**−5.55**	**12.08**
	Present to SSP5-8.5	BC	77.03	25.65	−51.38	73.20
		MI	40.51	23.41	−17.09	30.91
		MR	29.06	15.00	−14.06	16.26
		**Mean**	**48.87**	**21.35**	**−27.51**	**40.12**

**Table 2:** Mean altitude for present and future projections of *Ophrys insectifera* distribution. For the future, two time slices, 2070 (2061–2080) and 2090 (2081–2100), are presented for all selected global circulation models (GCMs) and two shared socioeconomic pathways (SSPs), representing ‘best’- and ‘worst’-case scenarios respectively. BC: BCC-CSM2-MR, MI: MIROC-ES2L, MR: MRI-ESM2-0. SSP1-2.6: ‘best’-case scenario, SSP5-8.5: ‘worst’-case scenario. The presented values are for the EnvThin ensemble model.

Time Slice	SSP	Period/GCM	Mean Altitude (m)
Present		Current	542.6
2070	SSP1-2.6	BC	484.7
		MI	520.3
		MR	467.5
	SSP5-8.5	BC	428.6
		MI	447.8
		MR	447.0
2090	SSP1-2.6	BC	504.6
		MI	505.0
		MR	498.3
	SSP5-8.5	BC	278.4
		MI	442.9
		MR	492.7

## Data Availability

Not applicable.

## Supplementary Materials

The following supporting information can be downloaded at: https://www.mdpi.com/article/10.3390/biology11040497/s1, Table S1: Predictors that did not show any collinearity issues and were included in the analysis; Table S2: Discrimination and calibration metrics for each one of the separate models (RF: random forest; BRT: boosted regression trees; BART: Bayesian additive regression trees; MaxEnt: maximum entropy), and for both occurrence data thinning procedures; Table S3: Evaluation metrics for the five selected methods for each ensemble model, and both occurrence data thinning procedures; Table S4: Five first most important variables for each of the separate models and for both occurrence data thinning procedures; Table S5: Percentages of area loss, gain, overall area change and current occurrences lost for future projections of *Ophrys insectifera* of the EnvThin ensemble for all selected GCMs and two SSPs (SSP2-4.5, SSP3-7.0); Table S6: Percentages of area loss, gain, overall area change and current occurrences lost for future projections of *Ophrys insectifera* of the GeoThin ensemble for all selected GCMs and all SSPs; Table S7: Mean altitude for present and future projections of *Ophrys insectifera* distribution for the EnvThin ensemble model for all selected GCMs and two SSPs (SSP2-4.5 and SSP3-7.0); Table S8: Mean altitude for present and future projections of *Ophrys insectifera* distribution for the GeoThin ensemble model for all selected GCMs and SSPs; Table S9: Distance matrices between current and future distribution centroids for the EnvThin ensemble, for all GCMs and SSPs; Table S10: Distance matrices between current and future distribution centroids for the GeoThin ensemble, for all GCMs and SSPs; Figure S1: Current habitat suitability map for *Ophrys insectifera*, for the ensemble model using the geographical thinning procedure; Figure S2: Current and future (2090) potential distribution maps for *Ophrys insectifera* EnvThin ensemble for all GCMs and two SSPs (SSP1-2.6 and SSP5-8.5) as ‘best’- and ‘worst’-case scenario; Figure S3: Current and future (2070) potential distribution maps for *Ophrys insectifera* EnvThin ensemble model for all GCMs and two SSPs (SSP2-4.5 and SSP3-7.0); Figure S4: Current and future (2090) potential distribution maps for *Ophrys insectifera* EnvThin ensemble model for all GCMs and two SSPs (SSP2-4.5 and SSP3-7.0); Figure S5: Current and future (2070) potential distribution maps for *Ophrys insectifera* GeoThin ensemble model for all GCMs and two SSPs (SSP1-2.6 and SSP5-8.5) as ‘best’ and ‘worst’ case scenario; Figure S6: Current and future (2090) potential distribution maps for *Ophrys insectifera* GeoThin ensemble model for all GCMs and two SSPs (SSP1-2.6 and SSP5-8.5) as ‘best’ and ‘worst’ case scenario; Figure S7: Current and future (2070) potential distribution maps for *Ophrys insectifera* GeoThin ensemble model for all GCMs and two SSPs (SSP2-4.5 and SSP3-7.0); Figure S8: Current and future (2090) potential distribution maps for *Ophrys insectifera* GeoThin ensemble model for all GCMs and two SSPs (SSP2-4.5 and SSP3-7.0); Figure S9: Median centroids for current and future (2090) projected distributions of *Ophrys insectifera*, for the EnvThin ensemble for all GCMs and two SSPs (SSP1-2.6 and SSP5-8.5); Figure S10: Median centroids for current and future (2070) projected distributions of *Ophrys insectifera*, for the EnvThin ensemble for all GCMs and two SSPs (SSP2-4.5 and SSP3-7.0); Figure S11: Median centroids for current and future (2090) projected distributions of *Ophrys insectifera*, for the EnvThin ensemble for all GCMs and two SSPs (SSP2-4.5 and SSP3-7.0); Figure S12: Median centroids for current and future (2070) projected distributions of *Ophrys insectifera*, for the GeoThin ensemble for all GCMs and two SSPs (SSP1-2.6 and SSP5-8.5); Figure S13: Median centroids for current and future (2090) projected distributions of *Ophrys insectifera*, for the GeoThin ensemble for all GCMs and two SSPs (SSP1-2.6 and SSP5-8.5); Figure S14: Median centroids for current and future (2070) projected distributions of *Ophrys insectifera*, for the GeoThin ensemble for all GCMs and two SSPs (SSP2-4.5 and SSP3-7.0); Figure S15: Median centroids for current and future (2090) projected distributions of *Ophrys insectifera*, for the GeoThin ensemble for all GCMs and two SSPs (SSP2-4.5 and SSP3-7.0).
