# Supplementary material for: ‘Fly to a Safer North’: Distributional Shifts of the Orchid Ophrys insectifera L. Due to Climate Change
Source: Biology (Basel). 2022 Mar 24;11(4):497. doi: 10.3390/biology11040497 (PMC9025215; doi:10.3390/biology11040497)
Supplement: Supplementary file 1 [file biology-11-00497-s001.zip › Supplementary Material - Figures.pdf]

Supplementary Material: Supplementary Figures **S1 – S15**

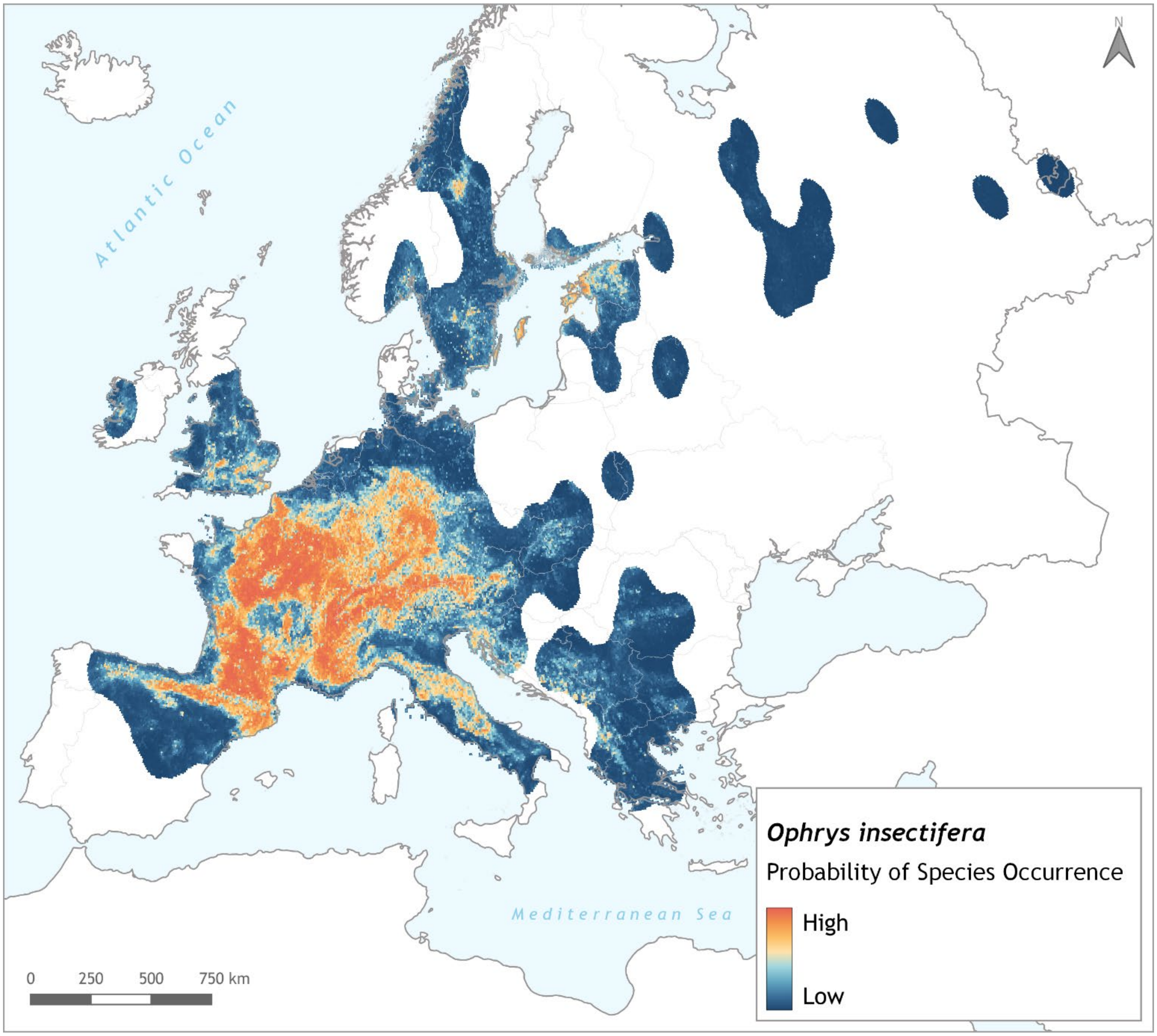

Figure S1. Current habitat suitability map for *Ophrys insectifera*, for the ensemble model using the geographical thinning procedure. Map is designed in QGIS v.3.18 'Zürich', using ETRS89 – Lambert Conformal Conic Coordinate Reference System.

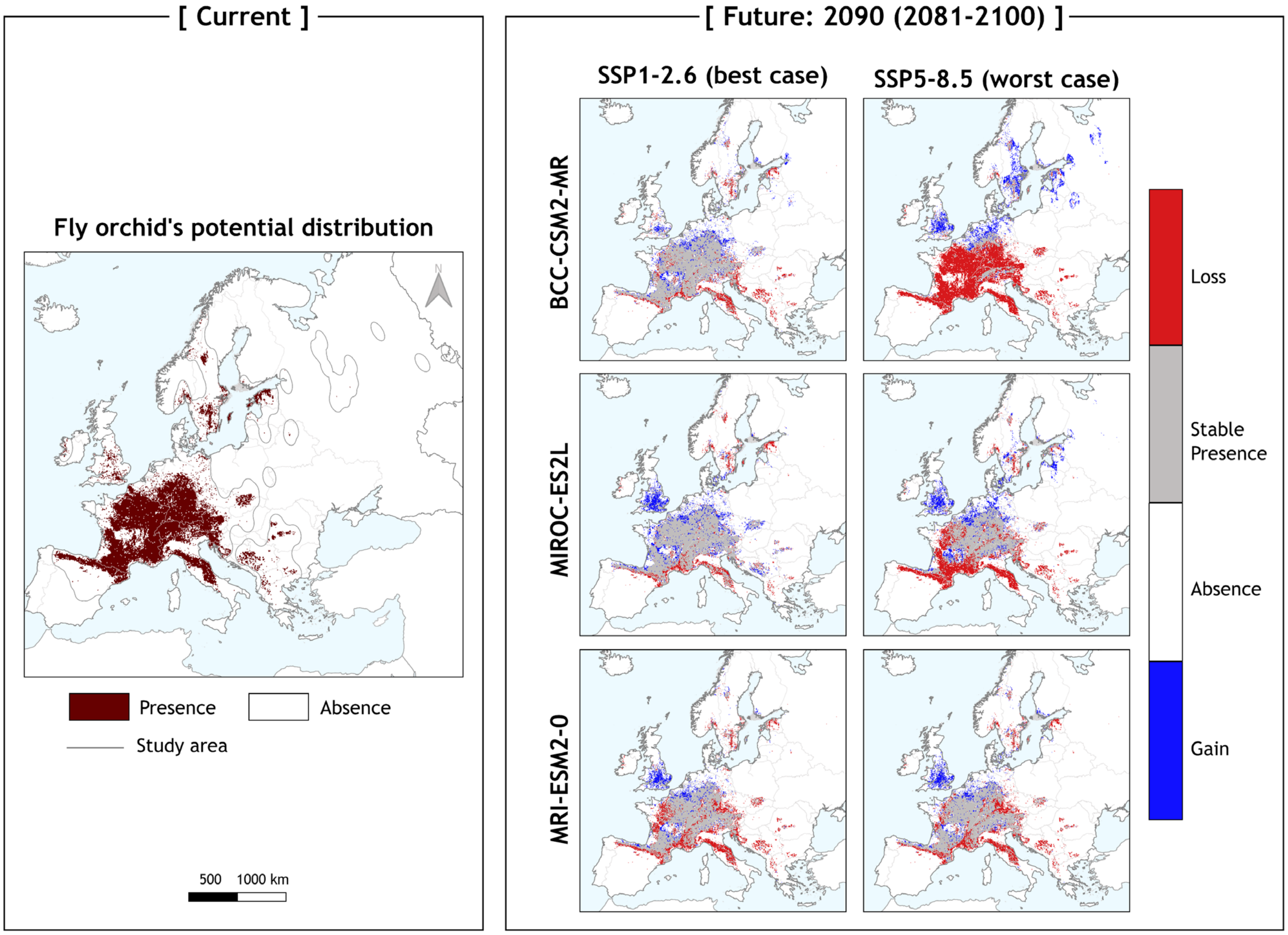

Figure S2. Current and future potential distribution maps for *Ophrys insectifera* EnvThin ensemble model. Left-hand panel: red-brown coloring indicates the cells the species currently potentially occupies. Each map is showing the transition from the present time-period to each respective GCM and SSP combination. Right-hand panel: future potential distribution maps of 2090 for the combinations of three GCMs (BCC, MIROC, and MRI) and two SSPs (SSP1-2.6 and SSP5-8.5) as 'best' and 'worst' case scenario. Grid cells with red colouring indicate the areas where the species is currently present but will not be in the future. Grey colouring represents cells where the species currently occupies and will continue to occupy in the future. White stands for the cells where the species is not currently present will not be in the future, while blue grid cells indicate the areas where the species is not currently present but will occupy in the future. All maps are designed in QGIS v.3.18 'Zürich', using ETRS89 – Lambert Conformal Conic Coordinate Reference System.

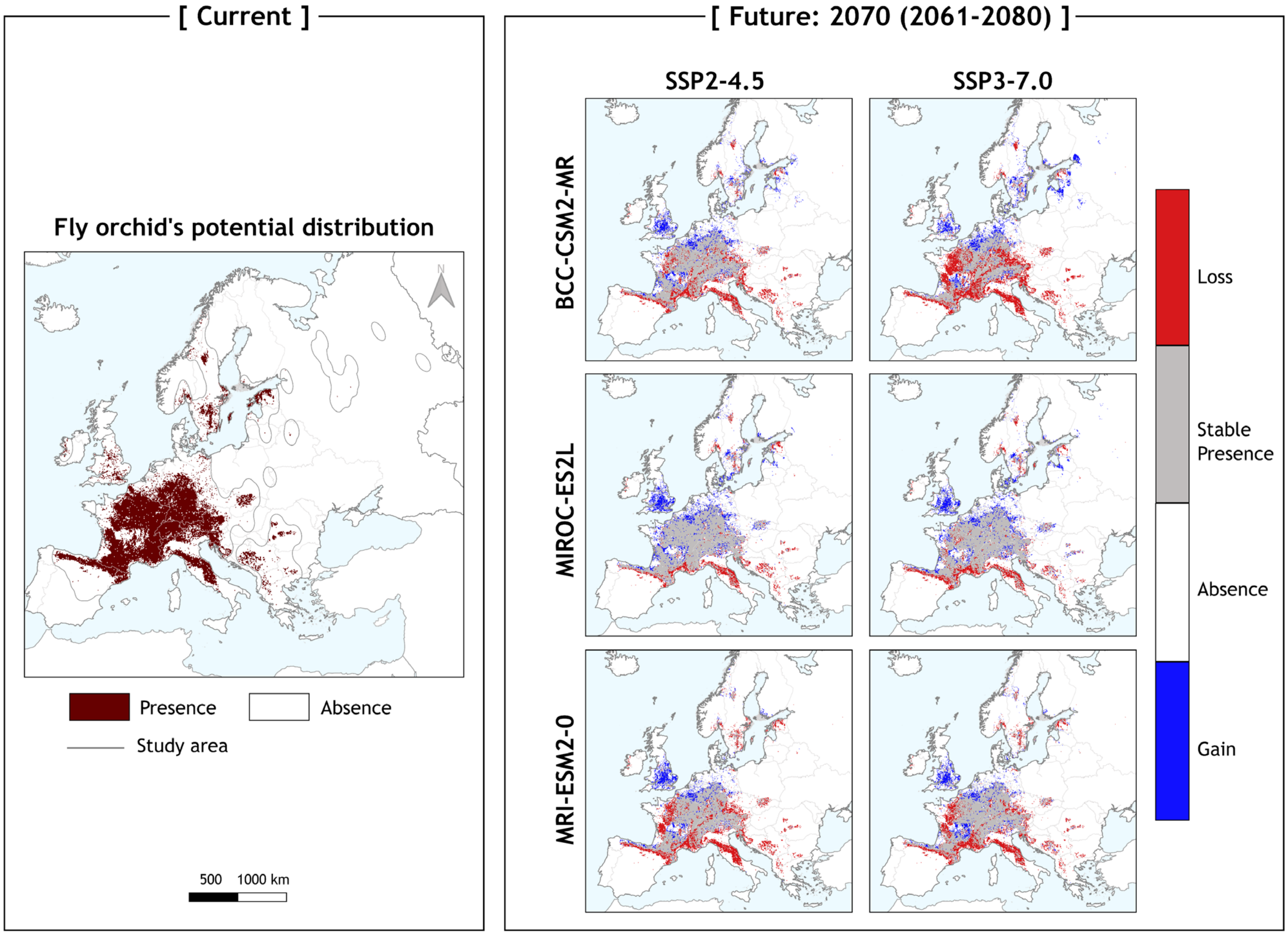

Figure S3. Current and future potential distribution maps for *Ophrys insectifera* EnvThin ensemble model. Left-hand panel: red-brown coloring indicates the cells the species currently potentially occupies. Each map is showing the transition from the present time-period to each respective GCM and SSP combination. Right-hand panel: future potential distribution maps of 2070 for the combinations of three GCMs (BCC, MIROC, and MRI) and two SSPs (SSP2-4.5 and SSP3-7.0). Grid cells with red colouring indicate the areas where the species is currently present but will not be in the future. Grey colouring represents cells where the species currently occupies and will continue to occupy in the future. White stands for the cells where the species is not currently present will not be in the future, while blue grid cells indicate the areas where the species is not currently present but will occupy in the future. All maps are designed in QGIS v.3.18 'Zürich', using ETRS89 – Lambert Conformal Conic Coordinate Reference System.

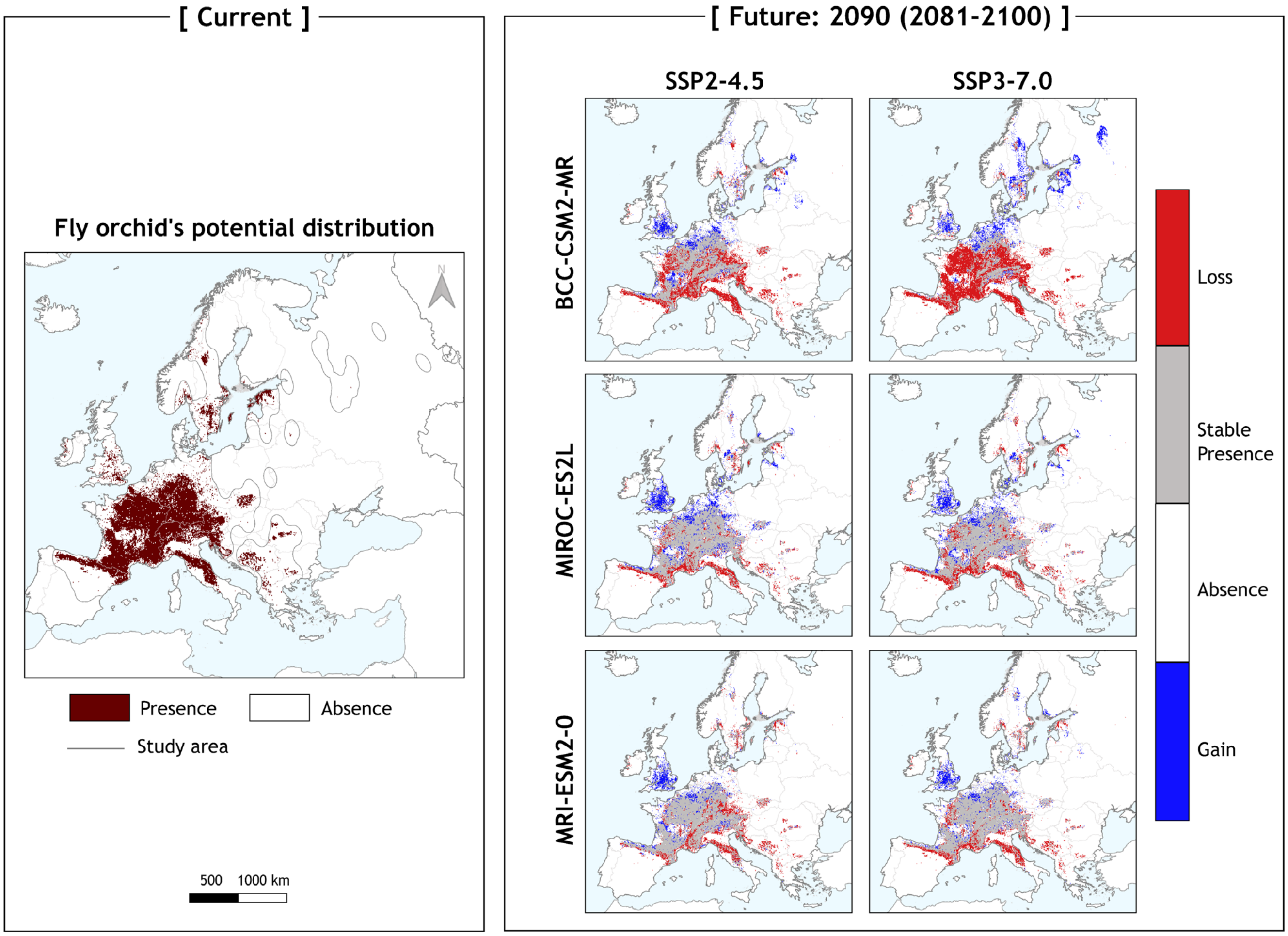

Figure S4. Current and future potential distribution maps for *Ophrys insectifera* EnvThin ensemble model. Left-hand panel: red-brown coloring indicates the cells the species currently potentially occupies. Each map is showing the transition from the present time-period to each respective GCM and SSP combination. Right-hand panel: future potential distribution maps of 2090 for the combinations of three GCMs (BCC, MIROC, and MRI) and two SSPs (SSP2-4.5 and SSP3-7.0). Grid cells with red colouring indicate the areas where the species is currently present but will not be in the future. Grey colouring represents cells where the species currently occupies and will continue to occupy in the future. White stands for the cells where the species is not currently present will not be in the future, while blue grid cells indicate the areas where the species is not currently present but will occupy in the future. All maps are designed in QGIS v.3.18 ‘Zürich’, using ETRS89 – Lambert Conformal Conic Coordinate Reference System.

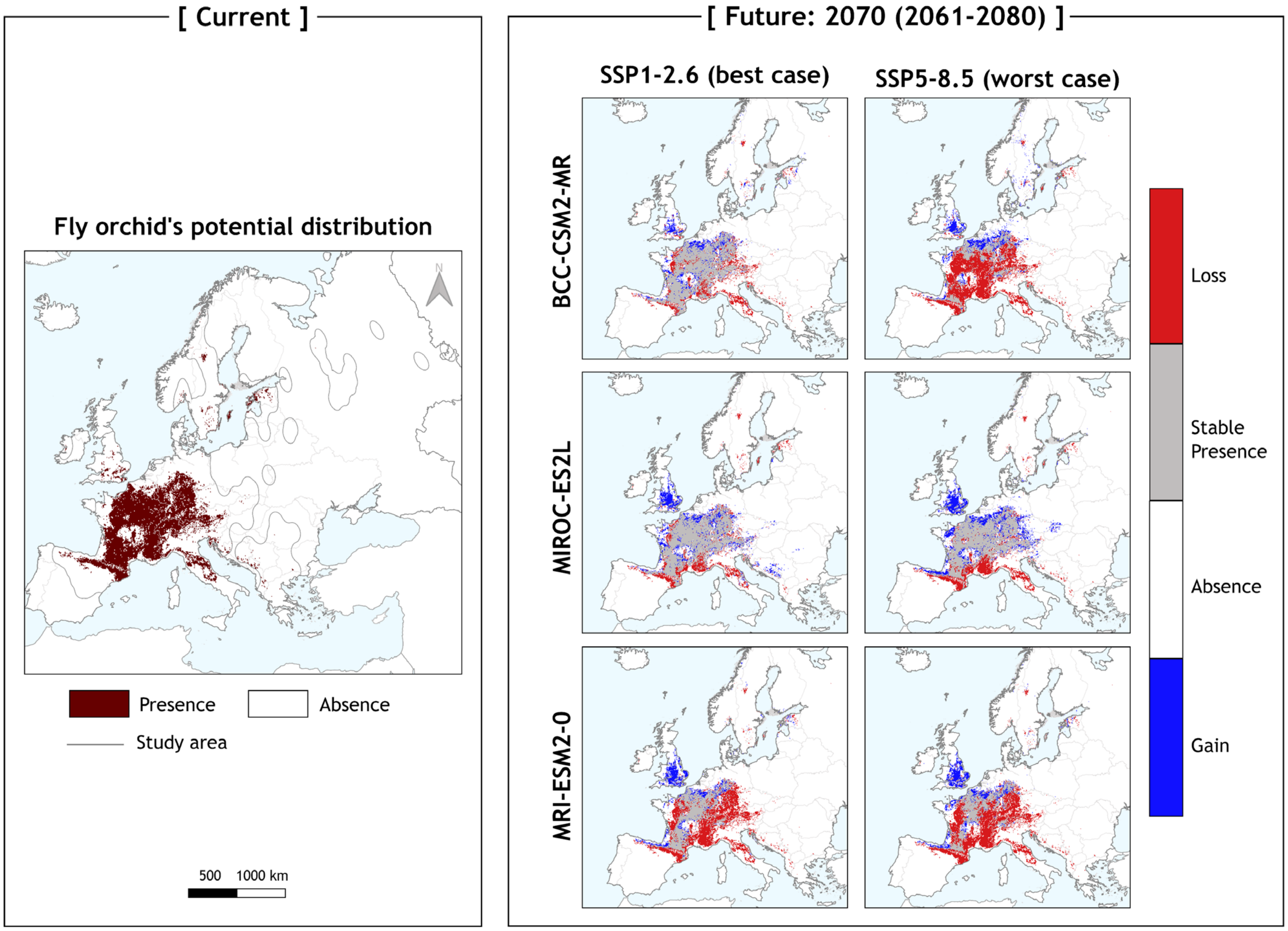

Figure S5. Current and future potential distribution maps for *Ophrys insectifera* GeoThin ensemble model. Left-hand panel: red-brown coloring indicates the cells the species currently potentially occupies. Each map is showing the transition from the present time-period to each respective GCM and SSP combination. Right-hand panel: future potential distribution maps of 2070 for the combinations of three GCMs (BCC, MIROC, and MRI) and two SSPs (SSP1-2.6 and SSP5-8.5) as 'best' and 'worst' case scenario. Grid cells with red colouring indicate the areas where the species is currently present but will not be in the future. Grey colouring represents cells where the species currently occupies and will continue to occupy in the future. White stands for the cells where the species is not currently present will not be in the future, while blue grid cells indicate the areas where the species is not currently present but will occupy in the future. All maps are designed in QGIS v.3.18 'Zürich', using ETRS89 – Lambert Conformal Conic Coordinate Reference System.

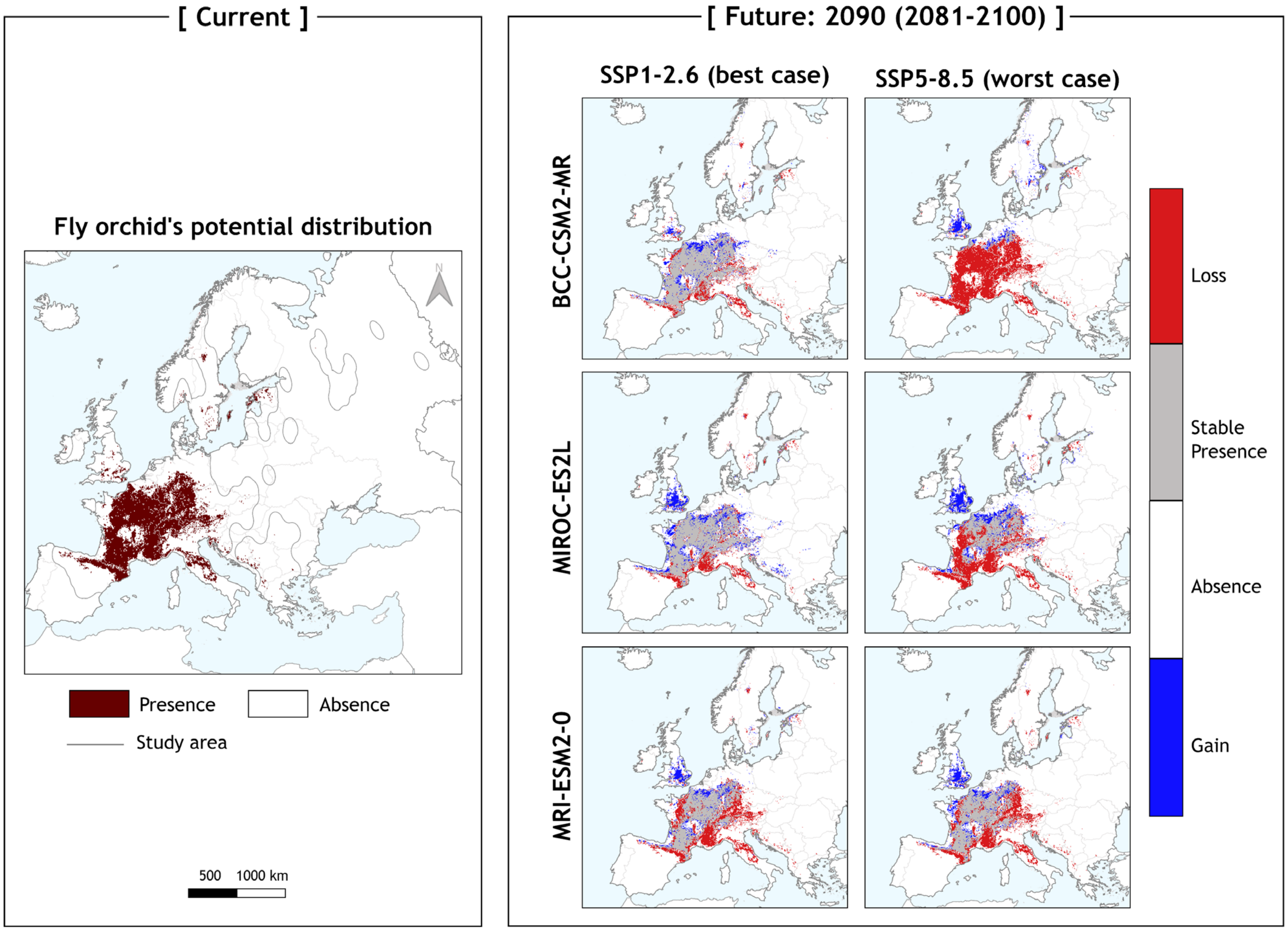

Figure S6. Current and future potential distribution maps for *Ophrys insectifera* GeoThin ensemble model. Left-hand panel: red-brown coloring indicates the cells the species currently potentially occupies. Each map is showing the transition from the present time-period to each respective GCM and SSP combination. Right-hand panel: future potential distribution maps of 2090 for the combinations of three GCMs (BCC, MIROC, and MRI) and two SSPs (SSP1-2.6 and SSP5-8.5) as 'best' and 'worst' case scenario. Grid cells with red colouring indicate the areas where the species is currently present but will not be in the future. Grey colouring represents cells where the species currently occupies and will continue to occupy in the future. White stands for the cells where the species is not currently present will not be in the future, while blue grid cells indicate the areas where the species is not currently present but will occupy in the future. All maps are designed in QGIS v.3.18 'Zürich', using ETRS89 – Lambert Conformal Conic Coordinate Reference System.

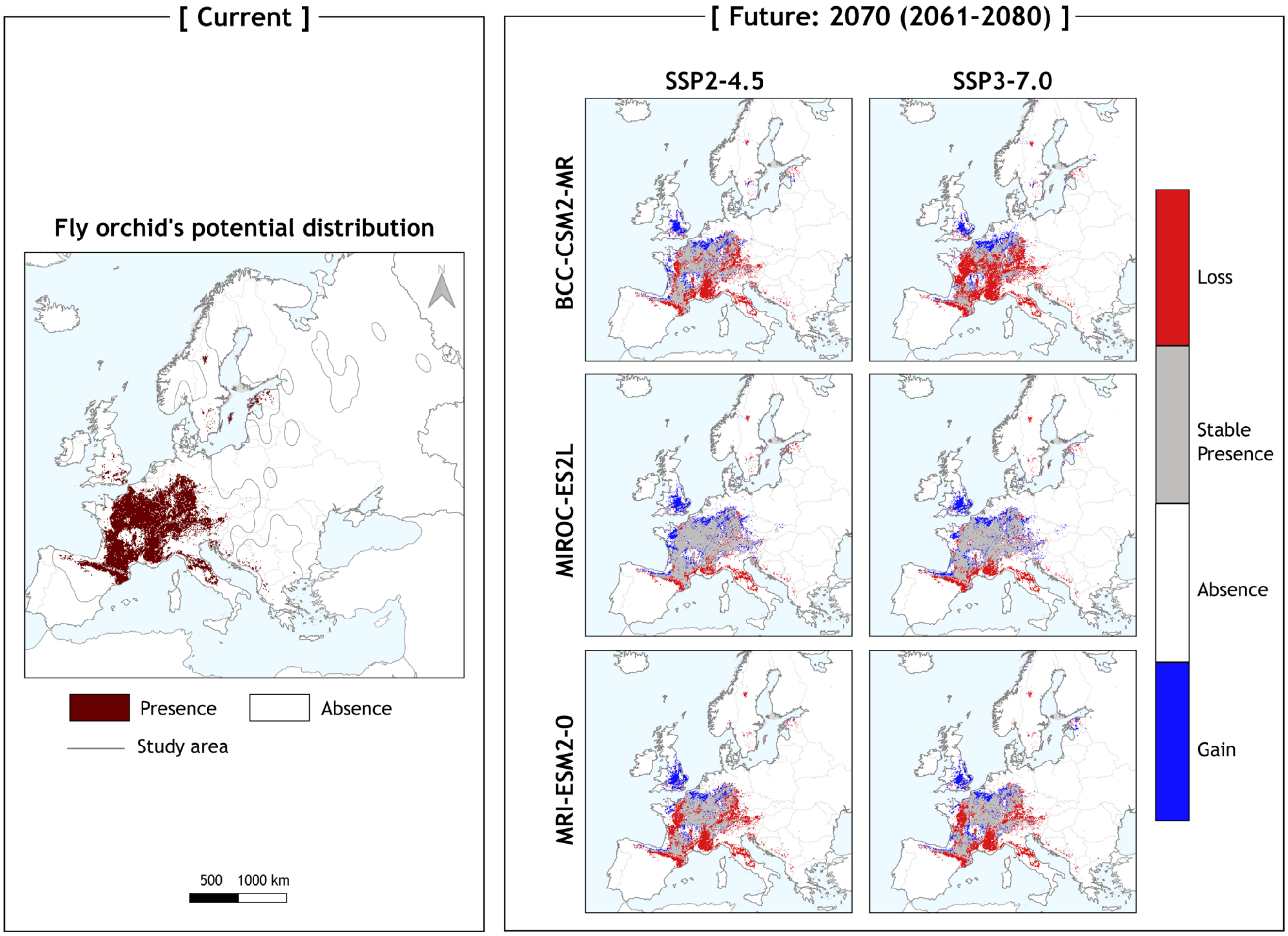

Figure S7. Current and future potential distribution maps for *Ophrys insectifera* GeoThin ensemble model. Left-hand panel: red-brown coloring indicates the cells the species currently potentially occupies. Each map is showing the transition from the present time-period to each respective GCM and SSP combination. Right-hand panel: future potential distribution maps of 2070 for the combinations of three GCMs (BCC, MIROC, and MRI) and two SSPs (SSP2-4.5 and SSP3-7.0). Grid cells with red colouring indicate the areas where the species is currently present but will not be in the future. Grey colouring represents cells where the species currently occupies and will continue to occupy in the future. White stands for the cells where the species is not currently present will not be in the future, while blue grid cells indicate the areas where the species is not currently present but will occupy in the future. All maps are designed in QGIS v.3.18 'Zürich', using ETRS89 – Lambert Conformal Conic Coordinate Reference System.

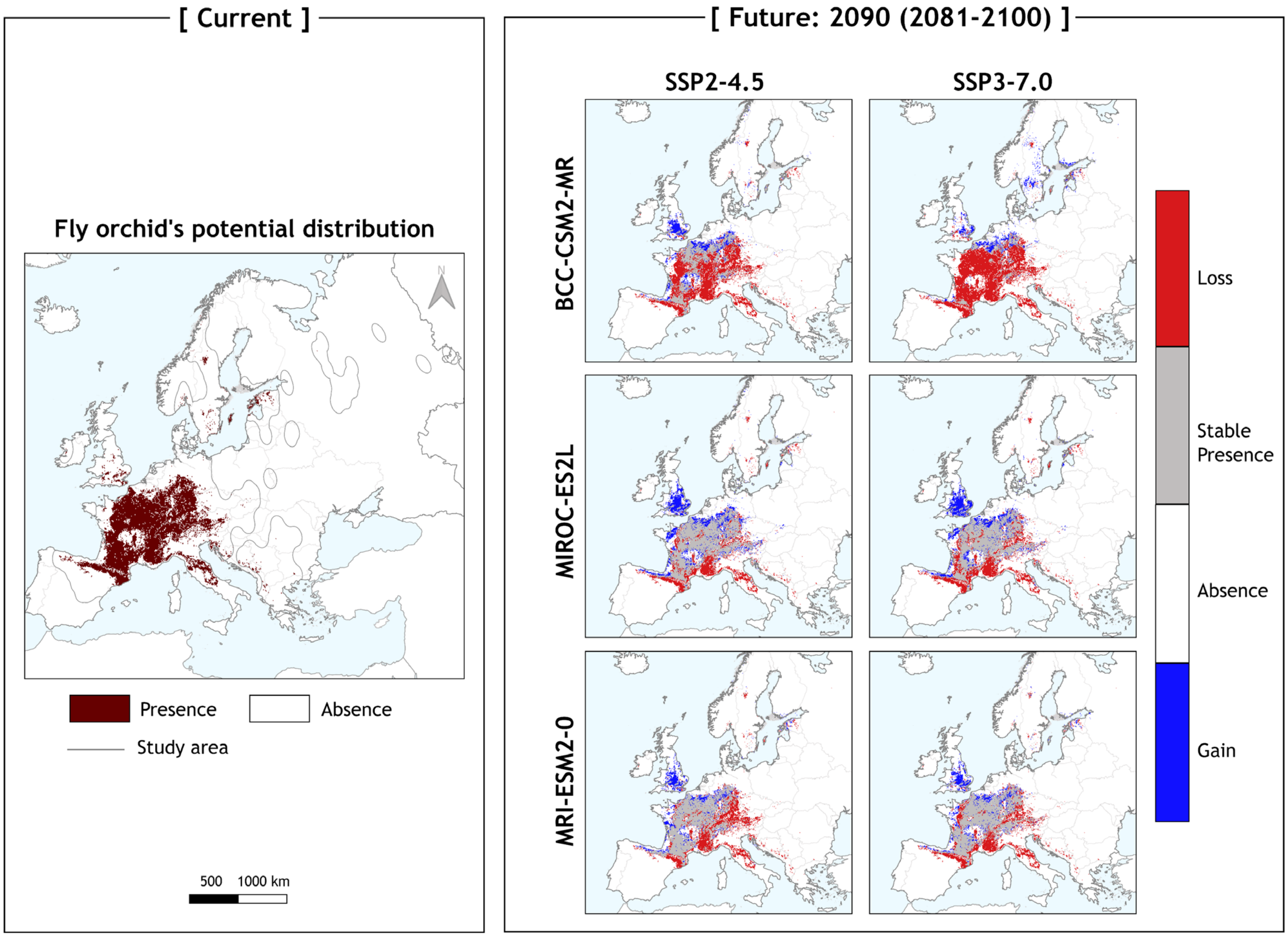

Figure S8. Current and future potential distribution maps for *Ophrys insectifera* GeoThin ensemble model. Left-hand panel: red-brown coloring indicates the cells the species currently potentially occupies. Each map is showing the transition from the present time-period to each respective GCM and SSP combination. Right-hand panel: future potential distribution maps of 2090 for the combinations of three GCMs (BCC, MIROC, and MRI) and two SSPs (SSP2-4.5 and SSP3-7.0). Grid cells with red colouring indicate the areas where the species is currently present but will not be in the future. Grey colouring represents cells where the species currently occupies and will continue to occupy in the future. White stands for the cells where the species is not currently present will not be in the future, while blue grid cells indicate the areas where the species is not currently present but will occupy in the future. All maps are designed in QGIS v.3.18 ‘Zürich’, using ETRS89 – Lambert Conformal Conic Coordinate Reference System.

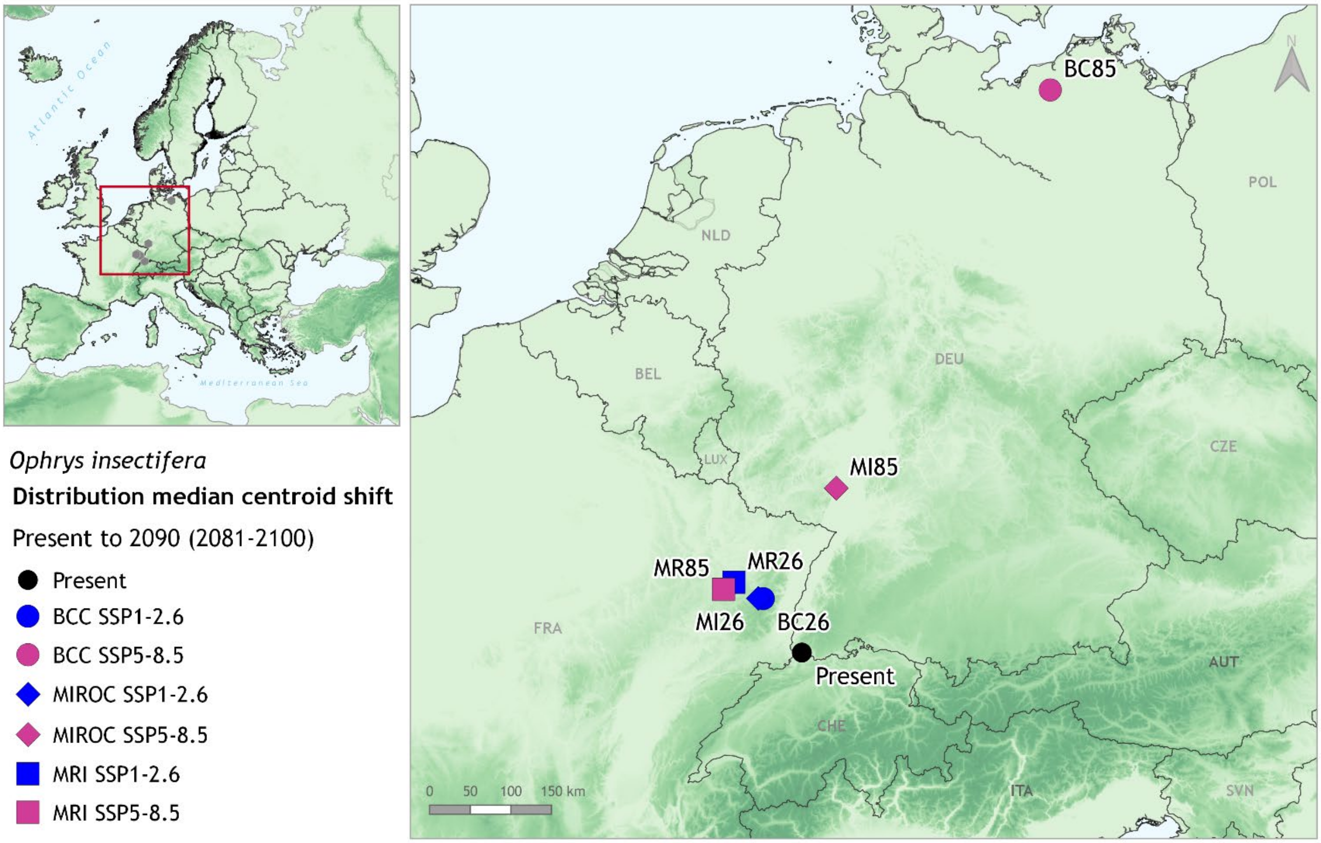

Figure S9. Median centroids for current and future projected distributions of *Ophrys insectifera*, for the EnvThin ensemble and 2090 timeslice. Black point represents the current distribution's median centroid, the blue points stand for the future best-case scenario (SSP1-2.6), and the magenta points for the future worst-case scenario (SSP5-8.5). GCMs differentiate by shape: BCC – circle, MIROC – diamond, MRI - square. Map is designed in QGIS v.3.18 'Zürich', using ETRS89 – Lambert Conformal Conic Coordinate Reference System.

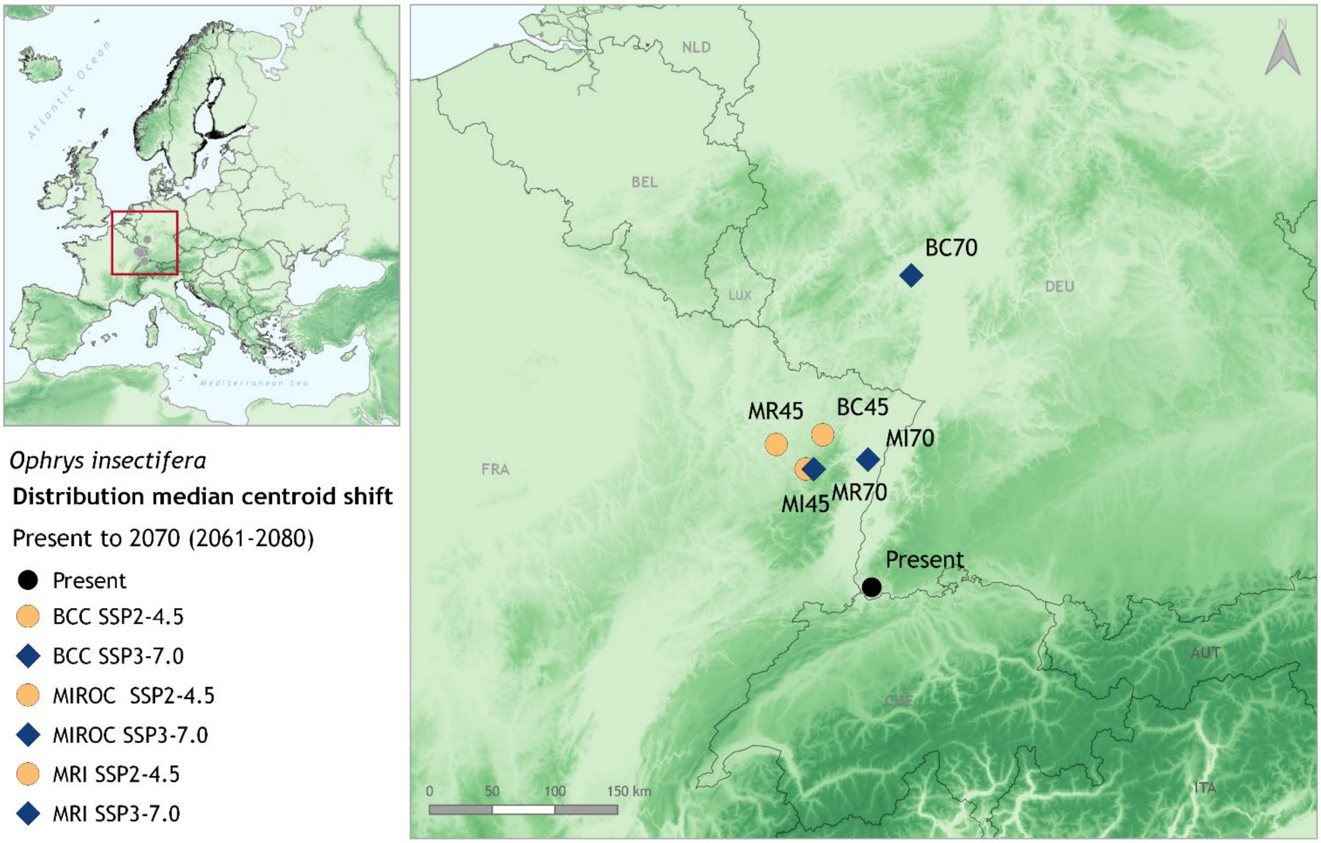

Figure S10. Median centroids for current and future projected distributions of *Ophrys insectifera*, for the EnvThin ensemble and 2070 timeslice. Black point represents the current distribution's median centroid, the yellow points stand for the future SSP2-4.5 scenario, and the navy blue points for the future SSP3-7.0 scenario. GCMs differentiate by shape: BCC – circle, MIROC – diamond, MRI - square. Map is designed in QGIS v.3.18 'Zürich', using ETRS89 – Lambert Conformal Conic Coordinate Reference System.

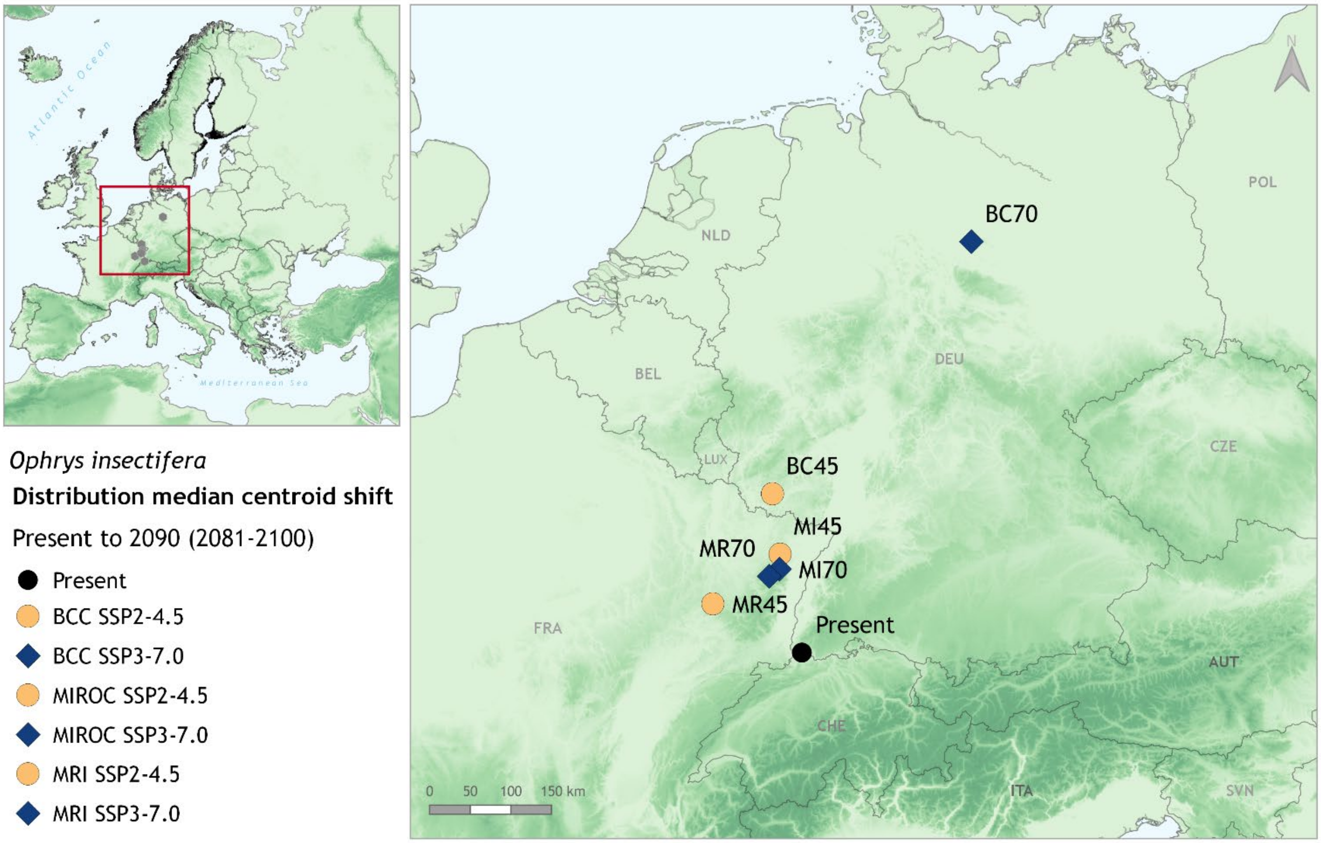

Figure S11. Median centroids for current and future projected distributions of *Ophrys insectifera*, for the EnvThin ensemble and 2090 timeslice. Black point represents the current distribution's median centroid, the yellow points stand for the future SSP2-4.5 scenario, and the navy blue points for the future SSP3-7.0 scenario. GCMs differentiate by shape: BCC – circle, MIROC – diamond, MRI - square. Map is designed in QGIS v.3.18 'Zürich', using ETRS89 – Lambert Conformal Conic Coordinate Reference System.

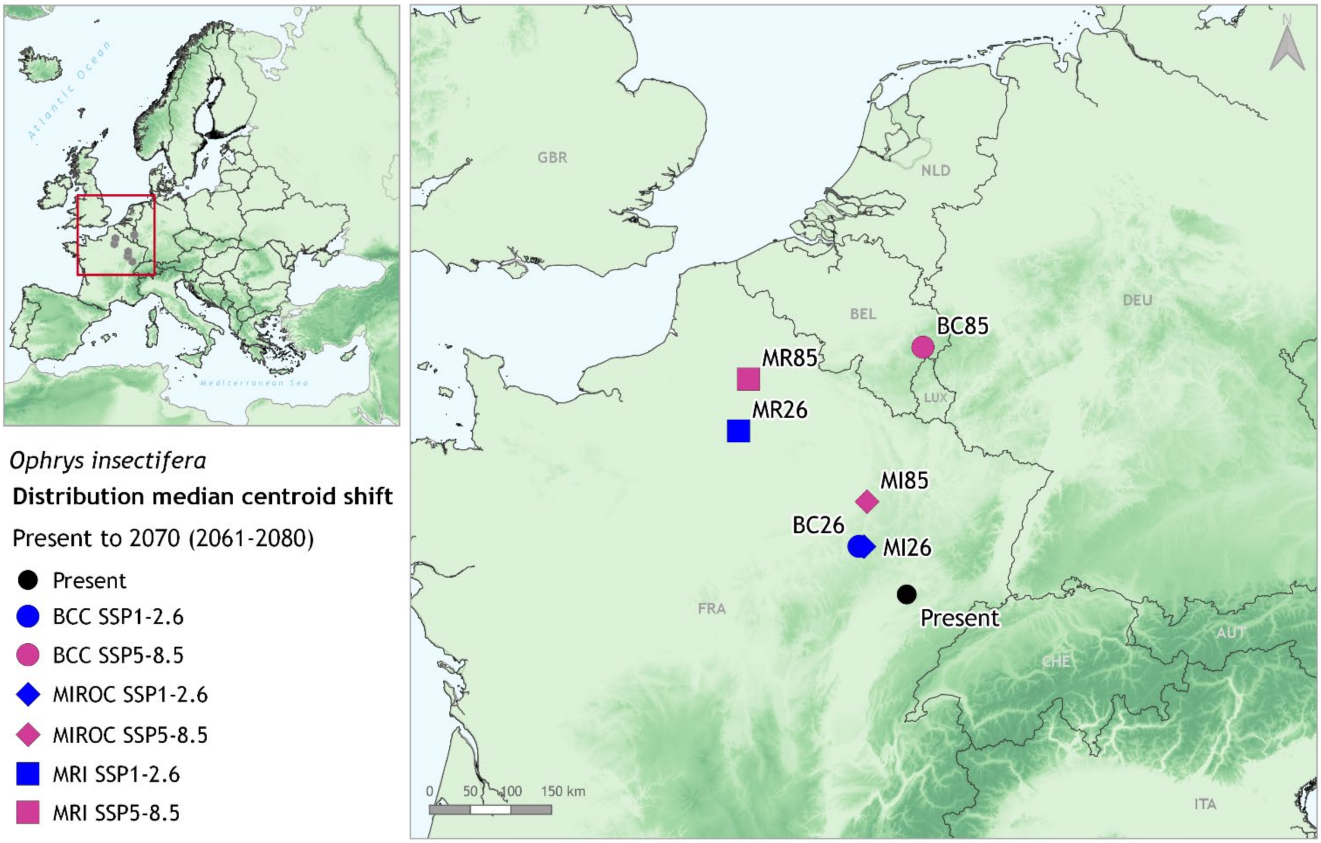

Figure S12. Median centroids for current and future projected distributions of *Ophrys insectifera*, for the GeoThin ensemble and 2070 timeslice. Black point represents the current distribution's median centroid, the blue points stand for the future best-case scenario (SSP1-2.6), and the magenta points for the future worst-case scenario (SSP5-8.5). GCMs differentiate by shape: BCC – circle, MIROC – diamond, MRI - square. Map is designed in QGIS v.3.18 'Zürich', using ETRS89 – Lambert Conformal Conic Coordinate Reference System.

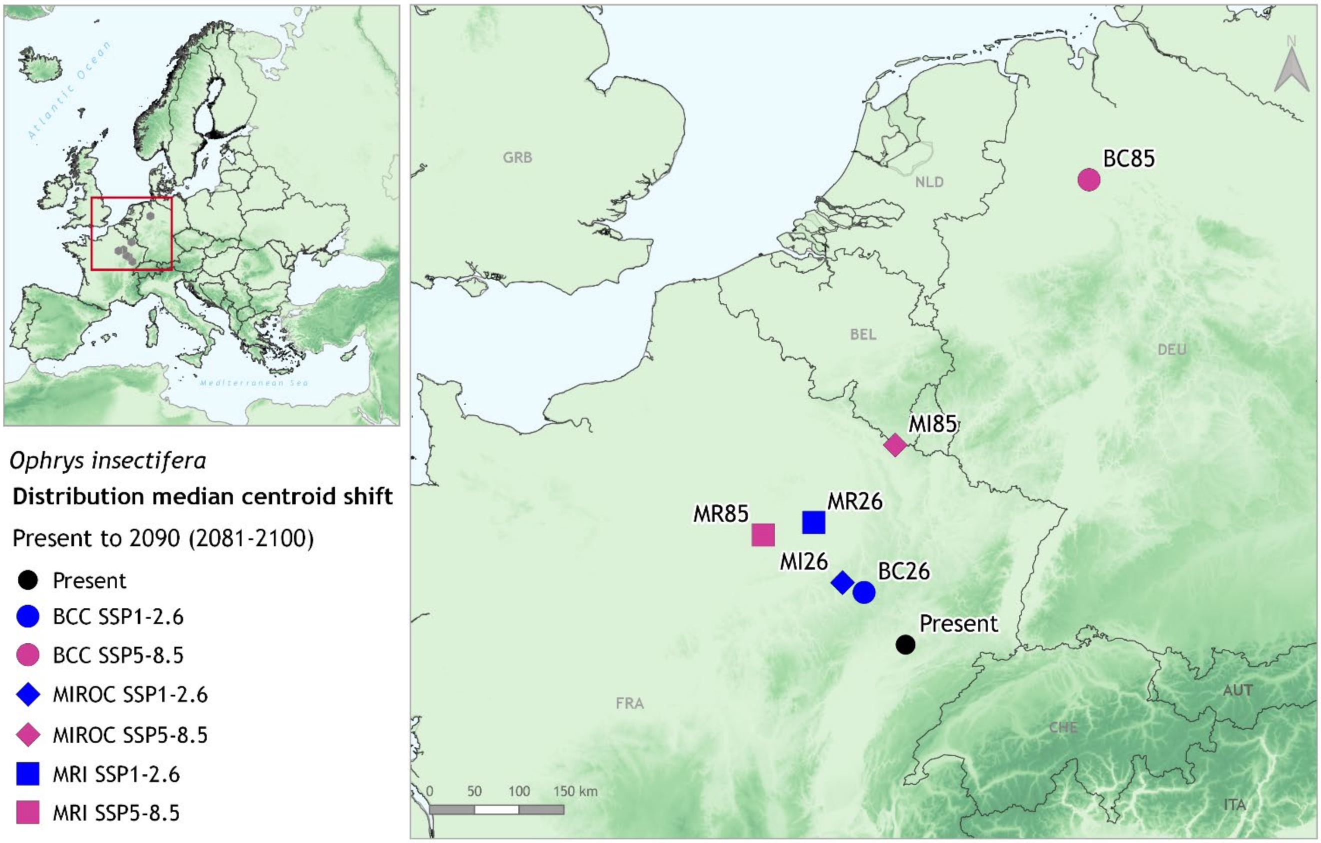

Figure S13. Median centroids for current and future projected distributions of *Ophrys insectifera*, for the GeoThin ensemble and 2090 timeslice. Black point represents the current distribution's median centroid, the blue points stand for the future best-case scenario (SSP1-2.6), and the magenta points for the future worst-case scenario (SSP5-8.5). GCMs differentiate by shape: BCC – circle, MIROC – diamond, MRI - square. Map is designed in QGIS v.3.18 'Zürich', using ETRS89 – Lambert Conformal Conic Coordinate Reference System.

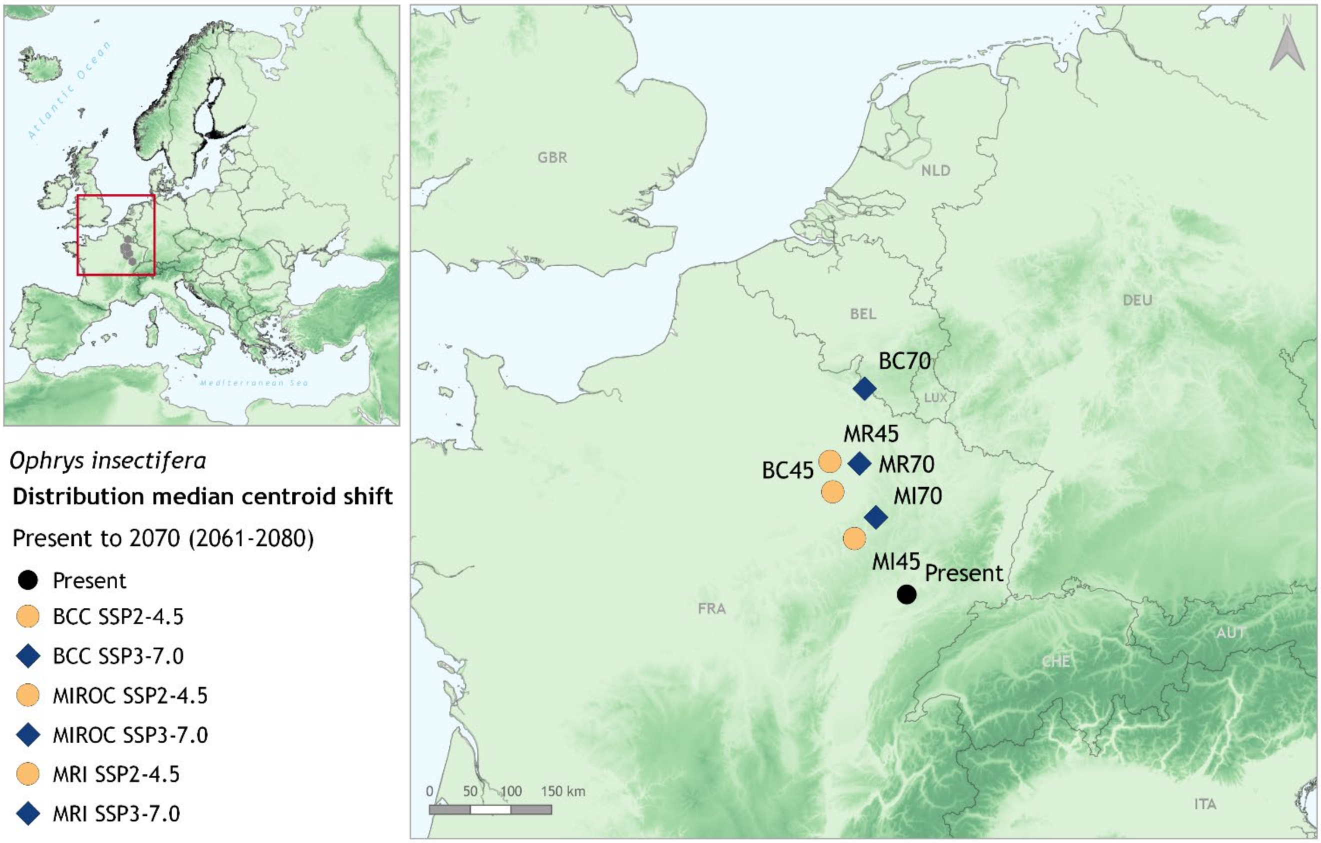

Figure S14. Median centroids for current and future projected distributions of *Ophrys insectifera*, for the GeoThin ensemble and 2070 timeslice. Black point represents the current distribution's median centroid, the yellow points stand for the future SSP2 4.5 scenario, and the navy blue points for the future SSP3 7.0 scenario. GCMs differentiate by shape: BCC – circle, MIROC – diamond, MRI - square. Map is designed in QGIS v.3.18 'Zürich', using ETRS89 – Lambert Conformal Conic Coordinate Reference System.

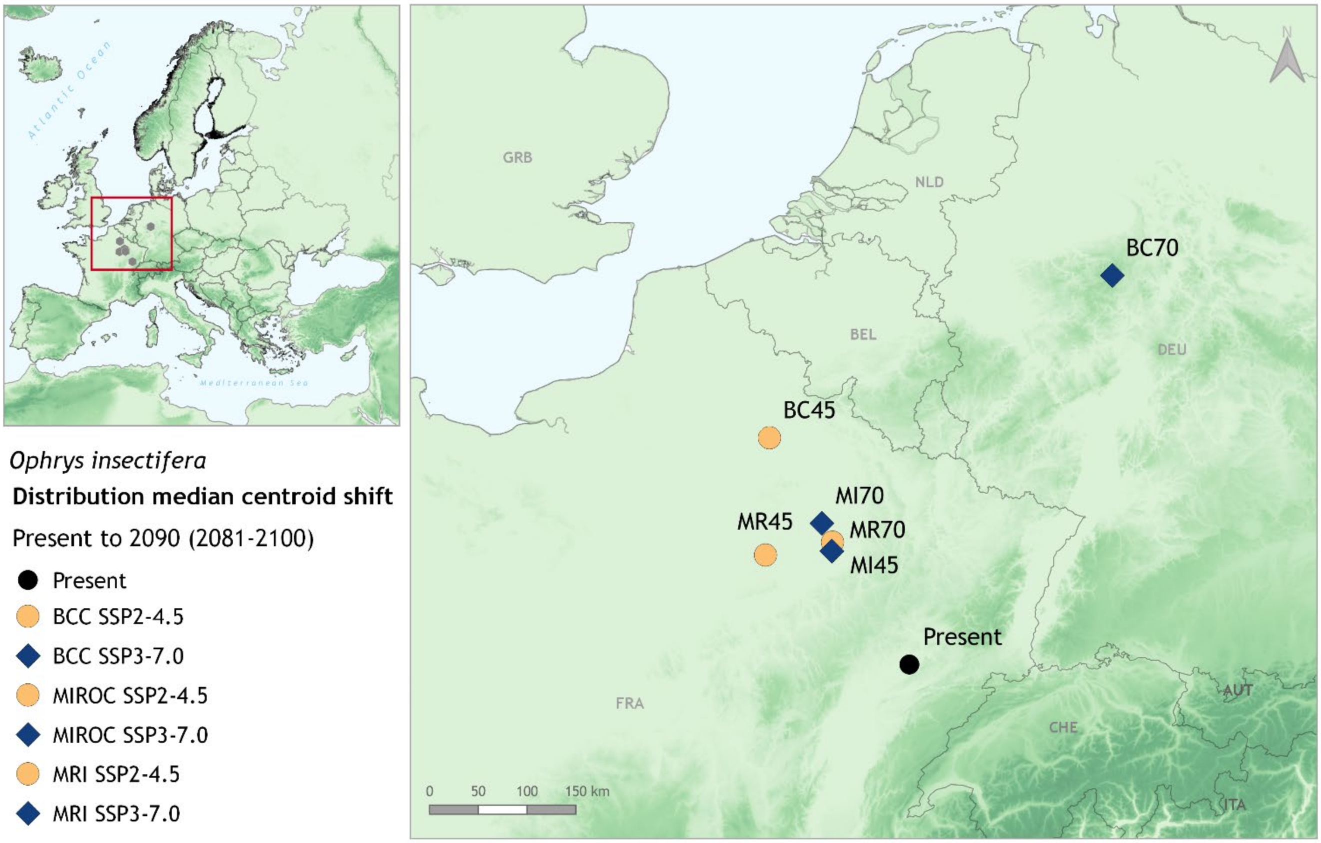

Figure S15. Median centroids for current and future projected distributions of *Ophrys insectifera*, for the GeoThin ensemble and 2090 timeslice. Black point represents the current distribution's median centroid, the yellow points stand for the future SSP2-4.5 scenario, and the navy blue points for the future SSP3-7.0 scenario. GCMs differentiate by shape: BCC – circle, MIROC – diamond, MRI - square. Map is designed in QGIS v.3.18 'Zürich', using ETRS89 – Lambert Conformal Conic Coordinate Reference System.
