# Supplementary material for: ‘Fly to a Safer North’: Distributional Shifts of the Orchid Ophrys insectifera L. Due to Climate Change
Source: Biology (Basel). 2022 Mar 24;11(4):497. doi: 10.3390/biology11040497 (PMC9025215; doi:10.3390/biology11040497)
Supplement: Supplementary file 1 [file biology-11-00497-s001.zip › Supplementary Material - Tables.pdf]

Table S1. Predictors that did not show any collinearity issues and were included in the analysis.

a/a	Variable code	Variable explanation
**1**	bio 2	Mean Diurnal Range (mean of monthly (max temp - min temp))
**2**	bio 4	Temperature Seasonality (standard deviation x 100)
**3**	bio 15	Precipitation Seasonality (Coefficient of Variation)
**4**	bio 19	Precipitation of Coldest Quarter
**5**	aridityIndT	Thornthwaite aridity index: index of the degree of water deficit below water need
**6**	mCByT10	Count of the number of months with Tmean > 10oC
**7**	PETDQ	Mean monthly potential evapotranspiration of driest quarter
**8**	PETWQ	Mean monthly potential evapotranspiration of wettest quarter
**9**	elev	Elevation data
**10**	slope	Slope (based on elevation data)
**11**	aspect	Aspect (based on elevation data)
**12**	HLI	Heat Load Index
**13**	TPI	Topographic Position Index
**14**	bdod	Mean bulk density of the fine earth fraction at a depth of 0-5cm
**15**	cfvo	Mean volumetric fraction of coarse fragments (>2mm) at a depth of 0-5cm
**16**	clay	Mean proportion of clay particles (<0.002mm) in the fine earth fraction at a depth of 0-5cm
**17**	nitrogen	Mean total nitrogen (N) at a depth of 0-5cm
**18**	sand	Mean proportion of sand particles (>0.05mm) in the fine earth fraction at a depth of 0-5cm
**19**	silt	Mean proportion of silt particles (≥0.002mm and ≤0.05mm) in the fine earth fraction at a depth of 0-5cm
**20**	soc	Mean soil organic carbon content in the fine earth fraction at a depth of 0-5cm

Table S2. Discrimination and calibration metrics for each one of the separate models (RF: Random Forest; BRT: Boosted Regression Trees; BART: Bayesian Additive Regression Trees; MaxEnt: Maximum Entropy), and for both occurrence data thinning procedures (EnvThin: environmental thinning; GeoThin: geographical thinning). 'Combined' refers to the mean of all models and thinning procedures. TSS: True Skill Statistic; AUC: Area under Curve; PR AUC: Area under Precision-Recall Curve; Kappa: Cohen's Kappa; CBI: Continuous Boyce Index; Sor: Sørensen's Index; Brier: Brier Score.

Thinning	Algorithm	TSS	AUC	PR AUC	Kappa	CBI	Sor	Brier
EnvThin	RF	0.98	1.00	1.00	0.86	0.99	0.78	0.01
	BRT	0.98	0.99	1.00	0.95	0.99	0.80	0.01
	BART	0.60	0.88	0.89	0.44	1.00	0.80	0.14
	MaxEnt	0.40	0.70	0.16	0.07	0.97	0.80	0.06
	Mean	**0.74**	**0.89**	**0.76**	**0.58**	**0.99**	**0.80**	**0.05**
	St. Dev.	0.29	0.14	0.40	0.41	0.01	0.01	0.06
GeoThin	RF	0.98	0.99	1.00	0.88	1.00	0.77	0.01
	BRT	0.93	0.99	0.99	0.85	1.00	0.80	0.03
	BART	0.66	0.90	0.90	0.55	1.00	0.80	0.12
	MaxEnt	0.46	0.70	0.32	0.18	1.00	0.80	0.09
	Mean	**0.76**	**0.90**	**0.80**	**0.61**	**1.00**	**0.79**	**0.07**
	SD	0.24	0.14	0.32	0.32	0.00	0.02	0.05
Combined	Mean	**0.747**	**0.895**	**0.782**	**0.598**	**0.994**	**0.794**	**0.059**
	SD	0.232	0.120	0.317	0.318	0.009	0.011	0.049

Table S3. Evaluation metrics for the five selected methods for each ensemble model, and both occurrence data thinning procedures (EnvThin: environmental thinning; GeoThin: geographical thinning). 'Combined' refers to the mean of both ensembles. Mean-P: mean of probabilities; Median-P: median of probabilities; wMean-P: weighted mean of probabilities; Com-Av: committee averaging of binary predictions; PCA: estimate of the PCA-weighted ensemble.

Thinning	Method	Sorensen	Brier	CBI	TSS	AUC
EnvEns	Mean-P	0.800	0.000	0.999	0.969	0.998
	Median-P	0.793	0.000	0.992	0.979	0.997
	wMean-P	0.800	0.000	1.000	0.980	0.999
	Com-Av	0.674	0.000	0.897	0.966	0.995
	PCA	0.800	0.000	0.998	0.963	0.997
	Mean	**0.773**	**0.000**	**0.977**	**0.971**	**0.997**
	StDev	0.056	0.000	0.045	0.008	0.001
GeoEns	Mean-P	0.800	0.000	1.000	0.910	0.991
	Median-P	0.799	0.000	0.999	0.930	0.993
	wMean-P	0.800	0.000	0.999	0.929	0.993
	Com-Av	0.677	0.000	0.897	0.940	0.988
	PCA	0.800	0.000	1.000	0.907	0.991
	Mean	**0.775**	**0.000**	**0.979**	**0.923**	**0.991**
	StDev	0.055	0.000	0.046	0.014	0.002
Combined	Mean	**0.774**	**0.000**	**0.978**	**0.947**	**0.994**
	StDev	0.052	0.000	0.043	0.028	0.003

Table S4. Five first most important variables for each of the separate models (RF: Random Forest; BRT: Boosted Regression Trees; BART: Bayesian Additive Regression Trees; MaxEnt: Maximum Entropy), and for both occurrence data thinning procedures (EnvThin: environmental thinning; GeoThin: geographical thinning). The variable importance values are scaled in a range of (0, 1), to be comparable. Variable codes (alphabetical order): aridityIndT – Thornthwaite aridity index; bio 4 – temperature seasonality (Standard Deviation x 100); bio 15 – precipitation seasonality (coefficient of variation); bio 19 – precipitation of coldest quarter; elev – elevation; PETDQ - Mean monthly potential evapotranspiration of driest quarter; PETWQ - Mean monthly potential evapotranspiration of wettest quarter.

Thinning	Algorithm	Variable code	Variable Importance (rescaled)
EnvThin	RF	bio 4	1.000
		elev	0.176
		sand	0.165
		aridityIndT	0.056
		bio 19	0.011
	BRT	bio 4	1.000
		aridityIndT	0.190
		PETDQ	0.062
		elev	0.058
		TPI	0.057
	BART	aridityIndT	1.000
		bio 4	0.901
		PETDQ	0.895
		PETWQ	0.665
		elev	0.535
GeoThin	RF	bio 4	1.000
		bio 15	0.392
		bio 19	0.236
		sand	0.162
		PETDQ	0.146
	BRT	bio 4	1.000
		elev	0.327
		aridityIndT	0.218
		bio 15	0.138
		sand	0.096
	BART	bio 4	1.000
		aridityIndT	0.744
		elev	0.454
		PETDQ	0.439
		PETWQ	0.415

Table S5. Percentages of area loss, gain, overall area change and current occurrences lost for future projections of Ophrys insectifera of the EnvThin ensemble. For the future, 2 time slices, 2070 (2061-2080) and 2090 (2081-2100), are presented for all selected GCMs (BC: BCC-CSM2-MR, MI: MIROC-ES2L, MR: MRI-ESM2-0) and two SSPs (SSP2-4.5, SSP3-7.0).

Thinning	Time slice	Transition	GCM	Area Loss (%)	Area Gain (%)	Overall Change (%)	Current occurrences lost (%)
EnvThin	2070	Present to SSP2 4.5	BC	27.75	20.02	-7.73	16.20
			MI	16.83	24.08	7.26	8.16
			MR	35.06	14.49	-20.57	22.03
			**Mean**	**26.55**	**19.53**	**-7.01**	**15.46**
		Present to SSP3 7.0	BC	45.3	21.55	-23.75	31.19
			MI	19.94	22.74	2.79	12.43
			MR	30.36	16.02	-14.34	18.65
			**Mean**	**31.87**	**20.10**	**-11.77**	**20.75**
	2090	Present to SSP2 4.5	BC	42.50	18.61	-23.89	29.13
			MI	22.98	25.01	2.03	14.10
			MR	24.27	14.89	-9.38	12.32
			**Mean**	**29.92**	**19.50**	**-10.41**	**18.52**
		Present to SSP3 7.0	BC	66.77	27.30	-39.47	56.83
			MI	27.20	21.33	-5.88	17.31
			MR	23.46	15.73	-7.73	11.93
			**Mean**	**39.14**	**21.45**	**-17.69**	**28.69**

Table S6. Percentages of area loss, gain, overall area change and current occurrences lost for future projections of Ophrys insectifera of the GeoThin ensemble. For the future, 2 time slices, 2070 (2061-2080) and 2090 (2081-2100), are presented for all selected GCMs (BC: BCC-CSM2-MR, MI: MIROC-ES2L, MR: MRI-ESM2-0) and all SSPs (SSP1-2.6, SSP2-4.5, SSP3-7.0 and SSP5-8.5).

Thinning	Time slice	Transition	GCM	Area Loss (%)	Area Gain (%)	Overall Change (%)	Current occurrences lost (%)
GeoThin	2070	Present to SSP1 2.6	BC	27.43	17.50	-9.93	21.41
			MI	24.51	25.60	1.09	20.90
			MR	56.90	19.30	-37.60	50.38
			**Mean**	**36.28**	**20.80**	**-15.48**	**30.90**
		Present to SSP2 4.5	BC	43.70	19.60	-24.10	37.74
			MI	22.70	26.60	3.86	17.55
			MR	47.40	19.80	-27.60	42.62
			**Mean**	**37.93**	**22.00**	**-15.95**	**32.63**
		Present to SSP3 7.0	BC	64.80	17.60	-47.20	60.30
			MI	27.10	26.70	-0.41	22.38
			MR	42.70	21.50	-21.20	38.76
			**Mean**	**44.87**	**21.93**	**-22.94**	**40.48**
		Present to SSP5 8.5	BC	70.70	20.50	-50.30	67.30
			MI	28.10	32.50	4.36	24.96
			MR	64.70	20.40	-44.30	58.00
			**Mean**	**54.50**	**24.47**	**-30.08**	**50.09**
	2090	Present to SSP1 2.6	BC	26.08	16.80	-9.28	20.16
			MI	25.13	27.00	1.87	21.82
			MR	44.10	16.80	-27.30	39.22
			**Mean**	**31.77**	**20.20**	**-11.57**	**27.07**
		Present to SSP2 4.5	BC	64.10	16.90	-47.10	58.69
			MI	32.02	29.10	-2.90	28.95
			MR	38.30	18.70	-19.60	33.34
			**Mean**	**44.81**	**21.57**	**-23.20**	**40.33**
		Present to SSP3 7.0	BC	86.30	16.01	-70.20	82.09
			MI	37.50	26.70	-10.80	33.90
			MR	35.10	18.70	-16.30	29.71
			**Mean**	**52.97**	**20.47**	**-32.43**	**48.43**
		Present to SSP5 8.5	BC	91.80	14.90	-76.90	86.97
			MI	54.30	27.10	-27.20	52.96
			MR	43.90	18.50	-25.40	38.17
			**Mean**	**63.33**	**20.17**	**-43.17**	**59.37**

Table S7. Mean altitude for present and future projections of *Ophrys insectifera* distribution for the EnvThin ensemble model. For the future, two time slices, 2070 (2061-2080) and 2090 (2081-2100), are presented for all selected GCMs (BC: BCC-CSM2-MR, MI: MIROC-ES2L, MR: MRI-ESM2-0) and two SSPs (SSP2-4.5 and SSP3-7.0).

Thinning	Timeslice	SSP	Period/GCM	Mean altitude (m)
EnvThin	Present		Current	542.6
	2070	SSP2 4.5	BC	465.9
			MI	473.3
			MR	487.4
		SSP3 7.0	BC	450.3
			MI	481.0
			MR	507.8
	2090	SSP2 4.5	BC	442.4
			MI	474.6
			MR	500.5
		SSP3 7.0	BC	387.5
			MI	478.1
			MR	489.9

Table S8. Mean altitude for present and future projections of *Ophrys insectifera* distribution for the GeoThin ensemble model. For the future, two time slices, 2070 (2061-2080) and 2090 (2081-2100), are presented for all selected GCMs (BC: BCC-CSM2-MR, MI: MIROC-ES2L, MR: MRI-ESM2-0) and SSPs (SSP1-2.6, SSP2-4.5, SSP3-7.0 and SSP5-8.5).

Thinning	Timeslice	SSP	Period/GCM	Mean altitude (m)
	Present		Current	504.9
			BC	432.4
			MI	456.5
			MR	276.1
		SSP2 4.5	BC	374.9
			MI	432.7
			MR	358.6
	2070*	SSP3 7.0	BC	347.7
			MI	418.8
			MR	385.0
		SSP5 8.5	BC	336.3
			MI	403.6
Geo			MR	257.1
		SSP1 2.6	BC	437.1
			MI	443.9
			MR	352.6
		SSP2 4.5	BC	324.6
			MI	401.6
			MR	360.0
	2090**	SSP3 7.0	BC	286.1
			MI	387.9
			MR	389.0
		SSP5 8.5	BC	155.5
			MI	363.3
			MR	362.6

* Kruskal Wallis test x2 = 1581.2, df = 6, $p < 0.001$

** Kruskal Wallis test x2 =998.7 , df = 6, $p < 0.001$

Table S9. Distance matrices between current and future distribution centroids for the EnvThin ensemble, for all GCMs and SSPs. BC: BCC-CSM2-MR, MI: MIROC-ES2L, MR: MRI-ESM2-0. 26: SSP1-2.6, 45: SSP2-4.5, 70: SSP3-7.0, 85: SSP5-8.5.

Ensemble	Timeslice	GCM/SSP	Present	BC26	BC45	BC70	BC85	MI26	MI45	MI70	MI85	MR26	MR45	MR70	MR85
EnvThin	2070	**Present**	0	104.0	128.0	251.4	323.5	59.6	108.2	102.1	129.7	140.5	137.4	105.2	186.6
		**BC26**	104.0	0	41.4	187.3	267.2	44.6	11.1	58.5	77.0	44.2	33.4	15.4	92.7
		**BC45**	128.0	41.4	0	145.9	225.8	74.4	30.4	41.3	43.9	67.5	38.0	28.5	58.5
		**BC70**	251.4	187.3	145.9	0	80.5	212.8	176.2	151.4	122.9	202.7	173.1	173.6	112.4
		**BC85**	323.5	267.2	225.8	80.5	0	290.1	256.1	227.1	198.9	283.1	253.6	253.1	191.6
		**MI26**	59.6	44.6	74.4	212.8	290.1	0	49.9	63.6	91.2	82.3	78.0	48.0	132.0
		**MI45**	108.2	11.1	30.4	176.2	256.1	49.9	0	50.1	66.6	49.3	30.8	6.2	83.6
		**MI70**	102.1	58.5	41.3	151.4	227.1	63.6	50.1	0	28.5	99.0	74.4	44.1	92.3
		**MI85**	129.7	77.0	43.9	122.9	198.9	91.2	66.6	28.5	0	110.8	81.9	61.6	76.6
		**MR26**	140.5	44.2	67.5	202.7	283.1	82.3	49.3	99.0	110.8	0	30.8	55.5	93.8
		**MR45**	137.4	33.4	38.0	173.1	253.6	78.0	30.8	74.4	81.9	30.8	0	35.9	67.4
		**MR70**	105.2	15.4	28.5	173.6	253.1	48.0	6.2	44.1	61.6	55.5	35.9	0	84.3
		**MR85**	186.6	92.7	58.5	112.4	191.6	132.0	83.6	92.3	76.6	93.8	67.4	84.3	0
	2090	**Present**	0	81.8	199.3	548.8	759.2	85.7	124.4	106.5	207.2	120.6	125.2	102.4	124.1
		**BC26**	81.8	0	129.9	510.1	721.0	6.2	58.6	41.4	163.3	41.5	62.7	28.5	50.4
		**BC45**	199.3	129.9	0	396.6	605.2	130.3	75.1	93.5	79.1	119.2	154.5	101.9	132.5
		**BC70**	548.8	510.1	396.6	0	211.0	513.1	452.5	468.7	347.2	512.7	549.5	482.8	527.2
		**BC85**	759.2	721.0	605.2	211.0	0	723.8	663.1	679.5	558.2	722.5	758.9	693.4	736.7
		**MI26**	85.7	6.2	130.3	513.1	723.8	0	60.8	44.5	166.6	36.0	56.6	30.4	44.3
		**MI45**	124.4	58.6	75.1	452.5	663.1	60.8	0	18.5	107.0	66.6	103.1	30.4	82.1
		**MI70**	106.5	41.4	93.5	468.7	679.5	44.5	18.5	0	122.0	58.5	92.7	15.4	73.3
		**MI85**	207.2	163.3	79.1	347.2	558.2	166.6	107.0	122.0	0	171.6	208.7	136.6	186.9
		**MR26**	120.6	41.5	119.2	512.7	722.5	36.0	66.6	58.5	171.6	0	37.2	44.2	15.4
		**MR45**	125.2	62.7	154.5	549.5	758.9	56.6	103.1	92.7	208.7	37.2	0	77.5	22.3
		**MR70**	102.4	28.5	101.9	482.8	693.4	30.4	30.4	15.4	136.6	44.2	77.5	0	58.6
		**MR85**	124.1	50.4	132.5	527.2	736.7	44.3	82.1	73.3	186.9	15.4	22.3	58.6	0

Table S10. Distance matrices between current and future distribution centroids for the GeoThin ensemble, for all GCMs and SSPs. BC: BCC-CSM2-MR, MI: MIROC-ES2L, MR: MRI-ESM2-0. 26: SSP1-2.6, 45: SSP2-4.5, 70: SSP3-7.0, 85: SSP5-8.5.

Ensemble	Time-slice	GCM/SSP	Present	BC26	BC45	BC70	BC85	MI26	MI45	MI70	MI85	MR26	MR45	MR70	MR85
GeoThin	2070	Present	0	83.6	156.1	259.2	305.8	79.0	94.4	102.4	124.5	289.3	189.4	171.6	329.7
		BC26	83.6	0	74.7	194.7	258.2	6.2	11.2	41.5	55.9	205.8	110.7	102.1	247.3
		BC45	156.1	74.7	0	133.3	210.5	78.0	63.6	62.0	44.0	138.3	37.6	48.1	173.6
		BC70	259.2	194.7	133.3	0	88.3	195.0	185.3	159.4	139.5	164.3	99.5	92.7	144.0
		BC85	305.8	258.2	210.5	88.3	0	256.8	250.9	217.9	202.9	250.1	181.9	163.7	219.1
		MI26	79.0	6.2	78.0	195.0	256.8	0	15.5	39.1	55.6	210.6	113.2	102.7	251.2
		MI45	94.4	11.2	63.6	185.3	250.9	15.5	0	37.2	48.0	195.1	99.8	92.7	236.2
		MI70	102.4	41.5	62.0	159.4	217.9	39.1	37.2	0	22.3	200.3	89.3	69.4	232.1
		MI85	124.5	55.9	44.0	139.5	202.9	55.6	48.0	22.3	0	180.9	67.5	47.9	210.4
		MR26	289.3	205.8	138.3	164.3	250.1	210.6	195.1	200.3	180.9	0	119.0	154.7	65.2
		MR45	189.4	110.7	37.6	99.5	181.9	113.2	99.8	89.3	67.5	119.0	0	36.6	143.0
		MR70	171.6	102.1	48.1	92.7	163.7	102.7	92.7	69.4	47.9	154.7	36.6	0	172.3
		MR85	329.7	247.3	173.6	144.0	219.1	251.2	236.2	232.1	210.4	65.2	143.0	172.3	0
	2090	Present	0	74.7	273.9	451.4	559.9	99.1	148.5	170.7	223.8	171.2	185.9	141.2	201.3
		BC26	74.7	0	199.9	426.6	526.6	26.5	74.7	96.9	168.6	96.5	115.0	66.8	130.0
		BC45	273.9	199.9	0	390.5	452.8	179.3	125.4	103.2	132.7	104.7	120.6	133.1	111.4
		BC70	451.4	426.6	390.5	0	120.7	433.1	397.8	393.0	282.7	408.2	458.5	404.7	461.2
		BC85	559.9	526.6	452.8	120.7	0	529.2	487.0	478.0	368.3	492.6	540.2	494.9	540.1
		MI26	99.1	26.5	179.3	433.1	529.2	0	57.0	78.1	165.0	74.7	88.5	48.0	103.6
		MI45	148.5	74.7	125.4	397.8	487.0	57.0	0	22.2	118.7	26.2	70.2	9.3	80.5
		MI70	170.7	96.9	103.2	393.0	478.0	78.1	22.2	0	110.8	15.4	66.6	30.4	73.0
		MI85	223.8	168.6	132.7	282.7	368.3	165.0	118.7	110.8	0	125.9	175.8	126.8	178.9
		MR26	171.2	96.5	104.7	408.2	492.6	74.7	26.2	15.4	125.9	0	51.2	30.8	58.3
		MR45	185.9	115.0	120.6	458.5	540.2	88.5	70.2	66.6	175.8	51.2	0	68.4	15.4
		MR70	141.2	66.8	133.1	404.7	494.9	48.0	9.3	30.4	126.8	30.8	68.4	0	80.0
		MR85	201.3	130.0	111.4	461.2	540.1	103.6	80.5	73.0	178.9	58.3	15.4	80.0	0
